# A Combination of Residual Distribution and the Active Flux Formulations or a New Class of Schemes That Can Combine Several Writings of the Same Hyperbolic Problem: Application to the 1D Euler Equations

**DOI:** 10.1007/s42967-021-00175-w

**Published:** 2022-03-23

**Authors:** R. Abgrall

**Affiliations:** Institute of Mathematics, University of Zürich, Winterthurerstrasse 190, CH 8057 Zurich, Switzerland

**Keywords:** Hyperbolic problems, high order, Active flux, MOOD, Residual distribution methods, 65M06, 65M08, 65M99

## Abstract

We show how to combine in a natural way (i.e., without any test nor switch) the conservative and non-conservative formulations of an hyperbolic system that has a conservative form. This is inspired from two different classes of schemes: the residual distribution one (Abgrall in Commun Appl Math Comput 2(3): 341–368, 2020), and the active flux formulations (Eyman and Roe in 49th AIAA Aerospace Science Meeting, 2011; Eyman in active flux. PhD thesis, University of Michigan, 2013; Helzel et al. in J Sci Comput 80(3): 35–61, 2019; Barsukow in J Sci Comput 86(1): paper No. 3, 34, 2021; Roe in J Sci Comput 73: 1094–1114, 2017). The solution is globally continuous, and as in the active flux method, described by a combination of point values and average values. Unlike the “classical” active flux methods, the meaning of the point-wise and cell average degrees of freedom is different, and hence follow different forms of PDEs; it is a conservative version of the cell average, and a possibly non-conservative one for the points. This new class of scheme is proved to satisfy a Lax-Wendroff-like theorem. We also develop a method to perform non-linear stability. We illustrate the behaviour on several benchmarks, some quite challenging.

## Introduction

The notion of conservation is essential in the numerical approximation of hyperbolic systems of conservation: if it is violated, there is no chance, in practice, to compute the right weak solution in the limit of mesh refinement. This statement is known since the celebrated work of Lax and Wendroff [20], and what happens when conservation is violated has been discussed by Hou and Le Floch [17]. This conservation requirement imposes the use of the conservation form of the system. However, in many practical situations, this is not really the one would like to deal with, since in addition to conservation constraints, one also seeks for the preservation of additional features, like contacts for fluid mechanics, or entropy decrease for shocks.

In this paper, we are interested in compressible fluid dynamics. Several authors have already considered the problem of the correct discretisation of the non-conservative form of the system. In the purely Lagrangian framework, when the system is described by the momentum equation and the Gibbs equality, this has been done since decades: one can consider the seminal work of Wilkins, to begin with, and the problem is still of interest; one can consider [5, 6, 10] where high order is sought for. In the case of the Eulerian formulation, there are less works. One can mention [4, 9, 16] where staggered meshes are used, the thermodynamic variables are localised in the cells, while the kinetic ones are localised at the grid points, or [3] where a non-conservative formulation with correction is used from scratch. The first two references show how to construct at most second-order scheme, while the last one shows this for any order. All constructions are quite involved in term of algebra, because one has to transfer information from the original grid and the staggered one.

In this paper, we aim at showing how the notion of conservation introduced in the residual distribution framework [1, 2, 23] is flexible enough to allow to deal directly with the non-conservative form of the system, while the correct solutions are obtained in the limit of mesh refinement. More precisely, we show how to deal with both the conservative and non-conservative forms of the PDE, without any switch, as it was the case in [19]. We illustrate our strategy on several versions of the non-conservative form, and provide first-, second and third-order accurate version of the scheme. More than a particular example, we describe a general strategy which is quite simple. The systems on which we will work are descriptions of the Euler equations for fluid mechanics.

- The conservation one:
  1 $$\begin{aligned} \frac{\partial }{\partial t}\left(\begin{array}{c}\rho \\ \rho u \\ E\end{array}\right)+\frac{\partial }{\partial x}\left(\begin{array}{c} \rho u\\ \rho u^2+p\\ u(E+p)\end{array}\right)=0 \end{aligned},$$
- the primitive formulation:
  2 $$\begin{aligned} \frac{\partial }{\partial t} \left(\begin{array}{c}\rho \\ u \\ p\end{array}\right)+\left(\begin{array}{c}\frac{\partial \rho u}{\partial x}\\ u\frac{\partial u}{\partial x}+\frac{1}{\rho }\frac{\partial p}{\partial x}\\ u\frac{\partial p}{\partial x}+(e+p)\frac{\partial u}{\partial x} \end{array}\right)=0 \end{aligned},$$
- the “entropy” formulation:
  3 $$\begin{aligned} \frac{\partial }{\partial t}\left(\begin{array}{c}p \\ u \\ s\end{array}\right)+\left(\begin{array}{c}u\frac{\partial p}{\partial x}+(e+p)\frac{\partial u}{\partial x}\\ u\frac{\partial u}{\partial x}+\frac{1}{\rho }\frac{\partial p}{\partial x}\\ u\frac{\partial s}{\partial x} \end{array}\right)=0 \end{aligned},$$

where as usual $\rho$ is the density, *u* the velocity, *p* the pressure, $E=e+\frac{1}{2}\rho u^2$ is the total energy, $e=(\gamma -1)p$ and $s=\log (p)-\gamma \log (\rho )$ is the entropy. The ratio of specific heats, $\gamma$ is supposed to be constant here, mostly for simplicity.

This paper has several sources of inspirations. The first one is the residual distribution (RD) framework, and in particular [1, 2, 23]. The second one is the family of active flux [7, 11–13, 15], where the solution is represented by a cell average and point values. The conservation is recovered from how the average is updated. Here the difference comes from the fact that in addition several forms of the same system can be conserved, as (1)–(3) for the point value update while a Lax-Wendroff like result can still be shown. If the same systems were used, both for the cell average and the point values, this would easily fit into the RD framework, using the structure of the polynomial reconstruction. The difference with the active flux is that we use only the representation of the solution within one cell, and not a fancy flux evaluation. Another difference is about the way the solution is evolved in time: the active flux method uses the method of characteristics to evolve the point value, while here we rely on more standard Runge-Kutta methods.

The format of the paper is as follows. In the first part, we explain the general principles of our method, and justify why, under the assumptions made on the numerical sequence for the Lax-Wendroff theorem (boundedness in $L^\infty$ and strong convergence in $L^p$, $p\geqslant 1$, of a subsequence toward a $v\in L^p$, then this *v* is a weak solution of the problem), we can also show the convergence of a subsequence to a weak solution of the problem, under the same assumptions. In the second part, we describe several discretisations of the method, and in the third part, we provide several simulations to illustrate the method.

In this paper, the letter *C* denotes a constant, and we use the standard “algebra”, for example $C\times C=C$, $C+C=C$, or $\alpha C=C$ for any constant $\alpha \in {\mathbb{R}}$.

## The Methods

### Principle

We consider the problem

4a $$\begin{aligned} \frac{\partial {\mathbf{u}}}{\partial t}+\frac{\partial {{\mathbf{f}}}({\mathbf{u}})}{\partial x}=0,\quad x\in {\mathbb{R}}\end{aligned}$$

with the initial condition

4b $$\begin{aligned} {\mathbf{u}}(x,0)={\mathbf{u}}_0(x),\quad x\in {\mathbb{R}}. \end{aligned}$$

Here ${\mathbf{u}}\in {\mathcal{D}}_{\mathbf{u}}\subset {\mathbb{R}}^p$. For smooth solutions, we also consider an equivalent formulation in the form

4c $$\begin{aligned} \frac{\partial {\mathbf{v}}}{\partial t}+J\frac{\partial {\mathbf{v}}}{\partial x}=0 \end{aligned},$$

where ${\mathbf{v}}=\Psi ({\mathbf{u}})\in {\mathcal{D}}_{\mathbf{v}}$ and $\Psi :{\mathcal{D}}_{\mathbf{u}}\rightarrow {\mathcal{D}}_{\mathbf{v}}$ is assumed to be one-to-one and $C^1$ (as well as the inverse function). For example, if (4) corresponds to (1), then

$$\begin{aligned} {\mathcal{D}}_{\mathbf{u}}=\left\{ (\rho , \rho u, E)\in {\mathbb{R}}^3 \text{ such that }\rho>0 \text{ and } E-\frac{1}{2}\rho u^2>0\right\} . \end{aligned}$$

If (4c) corresponds to (2), then

$$\begin{aligned} {\mathcal{D}}_{\mathbf{v}}=\{(\rho , u, p)\text{ such that }\rho>0 \text{ and } p>0\} \end{aligned}$$

and (for a perfect gas) the mapping $\Psi$ corresponds to $(\rho , \rho u, E)\mapsto \big(\rho , u, p=(\gamma -1)\big( E-\frac{1}{2}\rho u^2\big) \big),$ while

$$\begin{aligned} J=\left(\begin{array}{ccc} u &{} \rho &{}0\\ 0&{} u &{} \frac{1}{\rho }\\ 0&{} e+p &{} u \end{array}\right). \end{aligned}$$

For (3),

$$\begin{aligned} {\mathcal{D}}_{\mathbf{u}}=\{(p,u,s)\in {\mathbb{R}}^3, p>0 \}. \end{aligned}$$

More generally, we have $J=\big[\nabla _{\mathbf{u}}\big(\Psi ^{-1}\big)\big]\nabla _{\mathbf{u}}{{\mathbf{f}}}$.

The idea is to discretise simultaneously (4a) and (4c). Forgetting the possible boundary conditions, ${\mathbb{R}}$ is divided into non-overlapping intervals $K_{j+1/2}=[x_{j}, x_{j+1}],$ where $x_j<x_{j+1}$ for all $j\in {\mathbb{Z}}$. We set $\Delta _{j+1/2}=x_{j+1}-x_j$ and $\Delta =\max _j\Delta _{j+1/2}$. At the grid points, we will estimate ${\mathbf{v}}_j$ in time, while in the cells we will estimate the average value

$$\begin{aligned} \bar{\mathbf{u}}_{j+1/2}=\frac{1}{\Delta _{j+1/2}}\int _{x_j}^{x_{j+1}} {\mathbf{u}}(x) {\mathrm{d}}x. \end{aligned}$$

When needed, we have ${\mathbf{u}}_j=\Psi ^{-1}({\mathbf{v}}_j)$, however $\bar{\mathbf{v}}_{j+1/2}=\Psi (\bar{\mathbf{u}}_{j+1/2})$ is meaningless since the $\Psi$ does not commute with the average.

In $K_{j+1/2}$ any continuous function can be represented by ${\mathbf{u}}_j={\mathbf{u}}(x_j)$, ${\mathbf{u}}_{j+1}={\mathbf{u}}(x_{j+1})$, and $\bar{\mathbf{u}}_{j+1/2}$: one can consider the polynomial $R_{\mathbf{u}}$ defined on $K_{j+1/2}$ by

$$\begin{aligned} \big(R_{\mathbf{u}}\big)_{|K_{j+1/2}}(x)={\mathbf{u}}_j L_{j+1/2}^0+{\mathbf{u}}_{j+1}L_{j+1/2}^1+\bar{\mathbf{u}}_{j+1/2} L_{j+1/2}^{1/2} \end{aligned}$$

with

$$\begin{aligned} L_{j+1/2}^\xi (x)=\ell _\xi \left( \frac{x-x_{j}}{x_{j+1}-x_j}\right) \end{aligned}$$

and

$$\begin{aligned} \ell _0(s)=(1-s)(1-3s), \quad \ell _1(s)=s(3s-2), \qquad \ell _{1/2}(x)=6s(1-s). \end{aligned}$$

We see that

$$\begin{aligned}&\ell _0(0)=1, \quad \ell _0(1)=0, \quad \int _0^1 \ell _0(s) {\mathrm{d}}s=0,\\ &\ell _1(1)=1, \quad \ell _1(0)=0, \quad \int _0^1\ell _1(s) {\mathrm{d}}s=0,\\ &\ell _{1/2}(0)=0,\quad \ell _{1/2}(1)=0, \quad \int _0^1\ell _{1/2}(s) {\mathrm{d}}s=1. \end{aligned}$$

How to evolve $\bar{\mathbf{u}}_{j+1/2}$ following (4a) and $v_{j}$ following (4c) in time? The solution is simple for the average value: since

$$\begin{aligned} \Delta _{j+1/2} \; \frac{{\mathrm{d}}\bar{\mathbf{u}}_{j+1/2}}{{\mathrm{d}}t}+ {{\mathbf{f}}}({\mathbf{u}}_{j+1}(t))-{{\mathbf{f}}}({\mathbf{u}}_{j}(t)) =0, \end{aligned}$$

we simply take

5a $$\begin{aligned} \Delta _{j+1/2}\frac{{\mathrm{d}}\bar{\mathbf{u}}_{j+1/2}}{{\mathrm{d}}t}+\big( \hat{{\mathbf{f}}}_{j+1/2}-\hat{{\mathbf{f}}}_{j-1/2}\big)=0 \end{aligned},$$

where $\hat{{\mathbf{f}}}_{j+1/2}$ is a consistent numerical flux that depends continuously on its arguments. In practice, since the approximation is continuous, we take

5b $$\begin{aligned} \hat{{\mathbf{f}}}_{j+1/2}={{\mathbf{f}}}({\mathbf{u}}_j){={{\mathbf{f}}}\big(\Psi ^{-1}({\mathbf{v}}_j)\big).} \end{aligned}$$

For ${\mathbf{v}}$, we assume a semi-discrete scheme of the following form:

5c $$\begin{aligned} \frac{{\mathrm{d}}{\mathbf{v}}_j}{{\mathrm{d}}t}+ \overleftarrow{\Phi }^{\mathbf{v}}_{j+1/2}+\overrightarrow{\Phi }^{\mathbf{v}}_{j-1/2}=0 \end{aligned},$$

such that $\overleftarrow{\Phi }^{\mathbf{v}}_{j+1/2}+\overrightarrow{\Phi }^{\mathbf{v}}_{j+1/2}$ is a consistent approximation of $J\frac{\partial {\mathbf{v}}}{\partial x}$ in $K_{j+1/2}$. We will give examples later, for now we only describe the principles. In general the residuals $\overleftarrow{\Phi }^{\mathbf{v}}_{j+1/2}$ and $\overrightarrow{\Phi }^{\mathbf{v}}_{j-1/2}$ need to depend on some ${\mathbf{v}}_l$ and ${\mathbf{v}}_{l+1/2}\approx {\mathbf{v}}(x_{l+1/2})$. We can recover the missing information at the half points in the following steps.

(i) From ${\mathbf{v}}_j$, we can get ${\mathbf{u}}_j=\Psi ({\mathbf{v}}_j)$.
(ii) Then in $[x_{j}, x_{j+1}]$ we approximate ${\mathbf{u}}$ by
  $$\begin{aligned} R_{\mathbf{u}}(x)={\mathbf{u}}_j\ell _0\left( \frac{x-x_j}{\Delta _{j+1/2}}\right) +\bar{\mathbf{u}}_{j+1/2}\ell _{1/2}\left( \frac{x-x_j}{\Delta _{j+1/2}}\right) +{\mathbf{u}}_{j+1} \ell _1\left( \frac{x-x_j}{\Delta _{j+1/2}}\right) , \end{aligned}$$
  which enable to provide ${\mathbf{u}}_{j+1/2}:=R_{\mathbf{u}}(x_{j+1/2})$, i.e.,
  5d $$\begin{aligned} {\mathbf{u}}_{j+1/2}=\frac{3}{2} \bar{\mathbf{u}}_{j+1/2}-\frac{{\mathbf{u}}_j+{\mathbf{u}}_{j+1}}{4}. \end{aligned}$$
  Note that this relation is simply $\bar{\mathbf{u}}_{j+1/2}=\frac{1}{6}\big( {\mathbf{u}}_j+{\mathbf{u}}_{j+1}+4 {\mathbf{u}}_{j+1/2}\big),$ i.e., Simpson’s formula.
(iii) Finally, we state
  $$\begin{aligned} {\mathbf{v}}_{j+1/2}=\Psi ^{-1}(R_{\mathbf{u}}(x_{j+1/2})). \end{aligned}$$

In some situations, described later, we will also make the approximation

$$\begin{aligned} {\mathbf{v}}_{j+1/2}=\Psi ^{-1}(\bar{\mathbf{u}}_{j+1/2}), \end{aligned}$$

which is nevertheless consistent (but only first order accurate). As written above, the fluctuations $\overleftarrow{\Phi }^{\mathbf{v}}_{j+1/2}$ and $\overrightarrow{\Phi }^{\mathbf{v}}_{j+1/2}$ are functionals of the form $\Phi \big( \{{\mathbf{v}}_l, {\mathbf{v}}_{l+1/2}\}, j-p\leqslant l\leqslant j+p \big)$ for some fixed value of *p*. We will make the following assumptions.

(i) Lipschitz continuity: there exists *C* that depends only on ${\mathbf{u}}^0$ and *T* such that for any $j\in {\mathbb{Z}}$,
  6a $$\begin{aligned} \Vert \Phi ( \{{\mathbf{v}}_l, {\mathbf{v}}_{l+1/2}\}, j-p\leqslant l\leqslant j+p)\Vert \leqslant \frac{C}{\Delta _{j+1/2}}\left( \sum _{l=-p}^p \Vert {\mathbf{v}}_l-{\mathbf{v}}_{l+1/2}\Vert \right) . \end{aligned}$$
(ii) Consistency: set ${\mathbf{v}}^h=R_{\mathbf{u}}$, then
  6b $$\begin{aligned} \sum _{j\in {\mathbb{Z}}}\int _{K_{j+1/2}}\left\| \overleftarrow{\Phi }^{\mathbf{v}}_{j+1/2}+\overrightarrow{\Phi }^{\mathbf{v}}_{j+1/2} -J\frac{\partial {\mathbf{v}}^h}{\partial x}\right\|  {\mathrm{d}}{\mathbf{x}}\leqslant C \Delta . \end{aligned}$$
(iii) Regular mesh: the meshes are regular in the finite element sense.

The ODE systems (5) are integrated by a standard ODE solver. We will choose the Euler forward method, and the second order and third order SSP Runge-Kutta scheme.

### Analysis of the Method

In order to explain why the method can work, we will choose the simplest ODE integrator, namely the Euler forward method. The general case can be done in the same way, with more technical details. So we integrate (5) by

7 $$\begin{aligned} \bar{\mathbf{u}}_{j+1/2}^{n+1}=\bar{\mathbf{u}}_{j+1/2}^n-\frac{\Delta t_n}{\Delta _{j+1/2}} \big(\underbrace{ {{\mathbf{f}}}({\mathbf{u}}_{j+1}^n)-{{\mathbf{f}}}({\mathbf{u}}_j^n)}_{:=\delta _{j+1/2} {{\mathbf{f}}}}\big) \end{aligned}$$

and

8 $$\begin{aligned} {\mathbf{v}}_j^{n+1}={\mathbf{v}}_j^n -{\Delta t_n }\big( \overleftarrow{\Phi }^{\mathbf{v}}_{j+1/2}+\overrightarrow{\Phi }^{\mathbf{v}}_{j-1/2}\big). \end{aligned}$$

Setting $\Delta _j$ as the average of $\Delta _{j+1/2}$ and $\Delta _{j-1/2}$, we rewrite (8) as

9 $$\begin{aligned} {\mathbf{v}}_j^{n+1}={\mathbf{v}}_j^n -\frac{\Delta t_n }{\Delta _j}\delta _x{\mathbf{v}}_j \end{aligned}$$

and we note that, using the assumption (6a) as well as the fact that the mesh is shape regular, that there exists $C>0$ depending only on ${\mathbf{u}}^0$ and *T* such that

$$\begin{aligned} {\Vert \delta _x {\mathbf{v}}_j}\Vert \leqslant C \sum _{j=p-1}^{p+1} \Vert {\mathbf{v}}_j-{\mathbf{v}}_{j+1/2}\Vert . \end{aligned}$$

Using the transformation (5d), from (8), we can evaluate ${\mathbf{u}}_j^{n+1}=\Psi ({\mathbf{v}}_j^{n+1})$, and then write the update of ${\mathbf{u}}$ as

10 $$\begin{aligned} {\Delta _j \big( {\mathbf{u}}_j^{n+1}-{\mathbf{u}}_j^n\big) +\Delta t_n \delta _x{\mathbf{u}}_{j}=0}, \end{aligned}$$

where

$$\begin{aligned} \delta _x{\mathbf{u}}_{j}=\frac{\Delta _j}{\Delta t_n}\bigg( \Psi \bigg({\mathbf{v}}_j^n-\frac{\Delta t_n}{\Delta _j} \delta {\mathbf{v}}_j \bigg)-\Psi ({\mathbf{v}}_j^n) \bigg), \end{aligned}$$

which, thanks to the assumptions we have made on $\Psi$ satisfies

$$\begin{aligned} \Vert \delta _x {\mathbf{u}}_{j+1/2}\Vert \leqslant C \Vert \delta _x {\mathbf{v}}_j\Vert \leqslant C\sum _{j=-p}^{p}\Vert {\mathbf{v}}_{j+l}-{\mathbf{v}}_{j+l+1}\Vert \leqslant C \sum _{l=-p}^{p}\Vert {\mathbf{u}}_{j+l}-{\mathbf{u}}_{j+1+l}\Vert \end{aligned}$$

for some constants that depend on the gradient of $\Psi$ and the maximum of the ${\mathbf{v}}_i^n$ for $i\in {\mathbb{Z}}$.

To explain the validity of the approximation, we start by the Simpson formula, which is exact for quadratic polynomials:

$$\begin{aligned} \int _{x_{j}}^{x_{j+1}} f(x)  {\mathrm{d}}x\approx \frac{\Delta _{j+1/2}}{6}\big( f(x_j)+4 f(x_{j+1/2})+f(x_{j+1})\big). \end{aligned}$$

From the point values ${\mathbf{u}}_j$, ${\mathbf{u}}_{j+1}$, and ${\mathbf{u}}_{j+1/2}$ at times $t_n$ and $t_{n+1}$, we define the quadratic Lagrange interpolant $R_{{\mathbf{u}}^n}$ and $R_{{\mathbf{u}}^{n+1}}$ and then write

$$\begin{aligned}&\int _{x_{j}}^{x_{j+1}} { \varphi (x,t)} \big( R_{{\mathbf{u}}^{n+1}}-R_{{\mathbf{u}}^{n}}\big)  {\mathrm{d}}x\approx \frac{\Delta _{j+1/2}}{6}\big(\varphi _{j+1}({\mathbf{u}}_{j+1}^{n+1}-{\mathbf{u}}_{j+1}^n)\\ &\qquad \qquad \qquad \qquad \qquad \qquad \qquad+4\varphi _{j+1/2} ({\mathbf{u}}_{j+1/2}^{n+1}-{\mathbf{u}}_{j+1/2}^n)+ \varphi _{j}({\mathbf{u}}_{j}^{n+1}-{\mathbf{u}}_j^n)\big). \end{aligned}$$

Accuracy is not an issue here. Using (8) and (10), setting $\delta _j^{n+1/2}{\mathbf{u}}={\mathbf{u}}_j^{n+1}-{\mathbf{u}}_j^n$, we get

$$\begin{aligned}  \Sigma :=&\sum \limits _{[x_j, \,x_{j+1}], \,j\in {\mathbb{Z}}}\frac{\Delta _{j+1/2}}{6}\bigg( \varphi _{j+1}^n \delta _j^{n+1/2} {\mathbf{u}}+4\varphi _{j+1/2}^n \delta _{j+1/2}^{n+1/2}{\mathbf{u}}+ \varphi _{j}^n\delta _j^{n+1/2}{\mathbf{u}}\bigg)\\ =&\sum \limits _{[x_j, \,x_{j+1}], \,j\in {\mathbb{Z}}} \frac{\Delta _{j+1/2}}{6}\bigg(\varphi _{j+1}^n \delta _{j+1}^{n+1/2}{\mathbf{u}}\\ &+ 4\varphi _{j+1/2}^n\bigg( \frac{3}{2} \delta _{j+1/2}^{n+1/2} \bar{\mathbf{u}}-\frac{\delta _{j+1}^{n+1/2} {\mathbf{u}}+\delta _j^{n+1/2} {\mathbf{u}}}{4} \bigg)+ \varphi _{j}^n \delta _{j}^{n+1/2}{\mathbf{u}}\bigg)\\ =&\sum \limits _{[x_j, \,x_{j+1}], \,j\in {\mathbb{Z}}}\Delta _{j+1/2} \varphi _{j+1/2}^n\delta _{j+1/2}^{n+1/2}\bar{\mathbf{u}}\\ &+ \underbrace{\sum \limits _{j\in {\mathbb{Z}}} \frac{\delta _{j}^{n+1/2} {\mathbf{u}}}{6} \bigg(\Delta _{j+1/2}\big( \varphi _j-\varphi _{j+1/2}\big) +\Delta _{j-1/2}\big(\varphi _j-\varphi _{j-1/2}\big)\bigg)}_{S_n}.\end{aligned}$$

So that we get, using (10)

11 $$\begin{aligned} &\sum \limits _{n\in {\mathbb{N}}}\sum \limits _{[x_j, \,x_{j+1}], \,j\in {\mathbb{Z}}}\frac{\Delta _{j+1/2}}{6}\bigg( \varphi _{j+1}^n \delta _j^{n+1/2} {\mathbf{u}}+4\varphi _{j+1/2}^n \delta _{j+1/2}^{n+1/2}{\mathbf{u}}+ \varphi _{j}^n\delta _j^{n+1/2}{\mathbf{u}}\bigg)\\ & {-}\sum \limits _{n\in {\mathbb{N}}} \Delta t_n\sum \limits _{[x_j, \,x_{j+1}], \,j\in {\mathbb{Z}}} \varphi _{j+1/2}^n\delta _{j+1/2}{{\mathbf{f}}}\\ & -\sum \limits _{n\in {\mathbb{N}}} S_n=0.  \end{aligned}$$

Then using again (10) and the fact that the mesh is regular, we observe that

$$\begin{aligned} S_n= \Delta t_n \sum _j \Delta _j \; \delta _x{\mathbf{u}}_j+O(\Delta ^3). \end{aligned}$$

In Appendix A, we will show that in the limit, the contribution of the $S_n$ term will converges towards 0, while the first term of (11) will converge to

$$\begin{aligned} \int _0^{+\infty }\int _{\mathbb{R}}\frac{\partial \varphi }{\partial t}{\mathbf{u}} {\mathrm{d}}x {\mathrm{d}}t-\int _{\mathbb{R}}{\mathbf{u}}_0  {\mathrm{d}}x, \end{aligned}$$

while the second term will converge towards

$$\begin{aligned} \int _0^{+\infty }\int _{{\mathbb{R}}} \frac{\partial \varphi }{\partial x} {{\mathbf{f}}}({\mathbf{u}}) {\mathrm{d}}{\mathbf{x}}. \end{aligned}$$

This will be shown, using classical arguments, in Appendix A, so that we have

#### **Proposition 1**

*We assume that the mesh is regular*: *there exist* $\alpha$ *and* $\beta$ *such that* $\alpha \leqslant \Delta _{j+1/2}/\Delta _{j-1/2}\leqslant \beta .$ *If* $\max _{j\in {\mathbb{Z}}} \Vert {\mathbf{u}}_j^n\Vert _\infty$ *and* $\max _{j\in {\mathbb{Z}}} \Vert v_{j+1/2}^n\Vert _\infty$ *are bounded*,  *and a subsequence of* ${\mathbf{u}}_\Delta$ *converges in* $L^1$ *towards* ${\mathbf{u}},$ *then* ${\mathbf{u}}$ *is a weak solution of the problem*.

#### **Remark 1**

Indeed, the definition of a precise $\Delta _j$ is not really needed, and we come back to this in the next section. What is needed is a spatial scale that relates the updates in ${\mathbf{v}}$ and ${\mathbf{u}}$ in an incremental form of the finite difference type. This is why the assumption of mesh regularity is fundamental.

## Some Examples of Discretisation

We list possible choices: for $\frac{\partial {\mathbf{v}}}{\partial t}+J\frac{\partial {\mathbf{v}}}{\partial x}=0,$ where *J* is the Jacobian of ${{\mathbf{f}}}$ with respect to ${\mathbf{u}}$; they have been used in the numerical tests. The question here is to define $\overleftarrow{\Phi }_{j+1/2}$ and $\overrightarrow{\Phi }_{j+1/2}$ that are the contributions of $K_{j\pm 1/2}$ to $J\frac{\partial {\mathbf{v}}}{\partial x}$ so that

$$\begin{aligned} J\frac{\partial {\mathbf{v}}}{\partial x}(x_i)\approx \overleftarrow{\Phi }^{\mathbf{v}}_{j+1/2}+{\overrightarrow{\Phi }^{\mathbf{v}}_{j-1/2}}. \end{aligned}$$

We follow the work of Iserles [18] who gives all the possible schemes that guarantee a stable (in $L^2$) semi-discretisation of the convection equation, for a regular grid which we assume. The only difference in his notations and ours is that the grid on which are defined the approximation of the derivative is made of the mesh points $x_j$ and the half points $x_{j+1/2}$.

The first list of examples has an upwind flavour:

12 $$\begin{aligned} \overleftarrow{\Phi }^{\mathbf{v}}_{j+1/2}=\big({ J({\mathbf{v}}_j)}\big)^- \frac{\delta ^-_{j}{\mathbf{v}}}{\Delta _{j+1/2}/2}\quad \text{and}\quad \overrightarrow{\Phi }^{\mathbf{v}}_{j+1/2}=\big( J({\mathbf{v}}_{j+1})\big)^+\frac{\delta _{j+1}^+{\mathbf{v}}}{\Delta _{j+1/2}/2}, \end{aligned}$$

where $\delta ^\pm _j$ is an approximation of $\Delta _{j+1/2} \frac{\partial v}{\partial x}$ obtained from [18]^1^.

- First-order approximation: we take
  13 $$\begin{aligned} \delta _j^+{\mathbf{v}}={\mathbf{v}}_{j}-{\mathbf{v}}_{j-1/2}, \quad \delta _j^-{\mathbf{v}}={\mathbf{v}}_{j+1/2}-{\mathbf{v}}_j. \end{aligned}$$
- Second order: we take
  14 $$\left \{\begin{aligned}   \delta _j^-{\mathbf{v}}&=-\frac{3}{2}{\mathbf{v}}_j+2{\mathbf{v}}_{j+1/2}-\frac{{\mathbf{v}}_{j+1}}{2},\\ \delta _j^+{\mathbf{v}}&=\frac{{\mathbf{v}}_{j-1}}{2}-2{\mathbf{v}}_{j-1/2}+\frac{3}{2}{\mathbf{v}}_{j}. \end{aligned}\right.$$
- Third order: we take
  15 $$\left \{\begin{aligned}  {\delta _j^-=-\frac{v_{i+1}}{6}+v_{i+1/2}-\frac{v_i}{2}-\frac{v_{i-1/2}}{3}},\\ { \delta _j^+=\frac{v_{i-1}}{6}-v_{i-1/2}+\frac{v_i}{2}+\frac{v_{i+1/2}}{3}}. \end{aligned}\right.$$
- Fourth order: the fully centered scheme would be
  $$\begin{aligned} \delta _j^\pm {\mathbf{v}}={\frac{{\mathbf{v}}_{j+1}-{\mathbf{v}}_{j-1}}{12}+2\frac{{\mathbf{v}}_{j+1/2}-{\mathbf{v}}_{j-1/2}}{3}}, \end{aligned}$$
  but we prefer
  16 $$\left \{\begin{aligned}  \delta _j^-{\mathbf{v}}&=\frac{{\mathbf{v}}_{j-1/2}}{4}+\frac{5}{6}{\mathbf{v}}_j- \frac{3}{2}{\mathbf{v}}_{j+1/2}+\frac{{\mathbf{v}}_{j+1}}{2}-\frac{{\mathbf{v}}_{j+3/2}}{12},\\ \delta _j^+{\mathbf{v}}&=\frac{{\mathbf{v}}_{j+1/2}}{4}+\frac{5}{6}{\mathbf{v}}_j- \frac{3}{2}{\mathbf{v}}_{j-1/2}+\frac{1}{2}{\mathbf{v}}_{j-1}-\frac{{\mathbf{v}}_{j-3/2}}{12}. \end{aligned}\right.$$
- Etc $$\cdots $$

It can be useful to have more dissipative versions of a first-order scheme. We take

$$\begin{aligned} \bigg(J\frac{\partial v}{\partial x}\bigg)_j=\overleftarrow{\Phi }_{j+1/2}+\overrightarrow{\Phi }_{j-1/2} \end{aligned}$$

with

$$\begin{aligned}  \frac{\Delta _{j+1/2}}{2}\overleftarrow{\Phi }_{j+1/2}&=\frac{1}{2} \widehat{J\frac{\partial {\mathbf{v}}}{\partial x_j}}+\alpha \left({\mathbf{v}}_j-\frac{{\mathbf{v}}_j+{\mathbf{v}}_{j+1/2}}{2}\right),\\ \frac{\Delta _{j+1/2}}{2}\overrightarrow{\Phi }_{j+1/2}&=\frac{1}{2} \widehat{J\frac{\partial {\mathbf{v}}}{\partial x_{j+1}}}+\alpha \left({\mathbf{v}}_{j+1}- \frac{{\mathbf{v}}_{j+1}+{\mathbf{v}}_{j+1/2}}{2}\right), \end{aligned}$$

where $\widehat{J\frac{\partial v}{\partial x_l}}$ is a consistent approximation of $J\frac{\partial u}{\partial x}$ at $x_l$ and $\alpha$ is an upper-bound of the spectral radius of $J({\mathbf{v}}_j)$, $J({\mathbf{v}}_{j+1/2})$, and $J({\mathbf{v}}_{j+1})$. We take, for simplicity, ${\mathbf{v}}_{j+1/2}=\Psi ^{-1}(\bar{\mathbf{u}}_{j+1/2})$. For the model (2), we take

$$\begin{aligned} \frac{\Delta _{j+1/2}}{2}\widehat{J\frac{\partial v}{\partial x_j}}=\left(\begin{array}{c} (\rho u)_{j+1/2}-(\rho u)_j\\ \frac{1}{2} \big( u_{j+1/2}^2-u_j^2) + \frac{1}{{\tilde{\rho }}_{j+1/2}}\big( p_{j+1/2}-p_j\big)\\ {\tilde{u}}_{j+1/2}\big(p_{j+1/2}-p_j)+{\tilde{\rho c}^2}_{j+1/2}\big(u_{j+1/2}-u_j\big) \end{array}\right), \end{aligned}$$

where $${\tilde{\rho }}_{j+1/2}$$ is the geometric average of $$\rho _j$$ and $$\rho _{j+1/2}$$, $${\tilde{u}}_{j+1/2}$$ is the arithmetic average of $$u_j$$ and $$u_{j+1/2}$$, while ${\tilde{\rho c}^2}_{j+1/2}$. For the model (3), we take

$$\begin{aligned} \frac{\Delta _{j+1/2}}{2}\widehat{J\frac{\partial v}{\partial x_j}}=\left(\begin{array}{c} {\tilde{u}}_{j+1/2} \big( s_{j+1/2}-s_j\big)\\ \frac{1}{2} \big( u_{j+1/2}^2-u_j^2) + \frac{1}{{\tilde{\rho }}_{j+1/2}}\big( p_{j+1/2}-p_j\big)\\ {\tilde{u}}_{j+1/2}\big(p_{j+1/2}-p_j)+{\tilde{\rho c}^2}_{j+1/2}\big(u_{j+1/2}-u_j\big) \end{array}\right) . \end{aligned}$$

All this has a local Lax-Friedrichs’ flavour, and seems to be positivity preserving for the velocity and the pressure.

Using this, the method is

17a $$\begin{aligned} \frac{{\mathrm{d}}{\mathbf{v}}_j}{{\mathrm{d}}t}+\overleftarrow{\Phi }_{j+1/2}^{\mathbf{v}}+\overrightarrow{\Phi }_{j-1/2}^{\mathbf{v}}=0 \end{aligned}$$

combined with

17b $$\begin{aligned} \Delta x\frac{{\mathrm{d}}\bar{\mathbf{u}}_{j+1/2}}{{\mathrm{d}}t}+{{{\mathbf{f}}}({\mathbf{u}}_{j+1})-{{\mathbf{f}}}({\mathbf{u}}_j)}=0. \end{aligned}$$

We see in (17a) that the time derivative of ${\mathbf{v}}$ is obtained by adding two fluctuations, one computed for the interval $K_{j+1/2}=[x_j,x_{j+1}]$ and one for the interval $K_{j-1/2}=[x_{j-1},x_j]$. These fluctuations are obtained from (12) with the increments in ${\mathbf{v}}$ defined by (13), (15), (16), etc. In the sequel, we denote the scheme applied on the interval $K_{j+1/2}$ by $S_{j+1/2}(k)$ where the averages are integrated by (17b) and ${\mathbf{v}}$ by (17a) with the fluctuations (13) for $k=1$, (14) for $k=2$, and (15) for $k=3$, etc. To make sure that the first-order scheme is positivity preserving (at least experimentally), we may also consider the case denoted by $k=0,$ where $S_{j+1/2}(0)$ is the local Lax-Friedrichs scheme defined above. Both fluctuation (13) and the local Lax-Friedrichs scheme are first-order accurate, but the second one is quite dissipative but positivity preserving while the scheme (13) is not (experimentally) positivity preserving. The system (17) is integrated in time by a Runge-Kutta solver: RK1, RK SSP2, and RK SSP3.

### Error Analysis in the Scalar Case

**Fig. 1:**
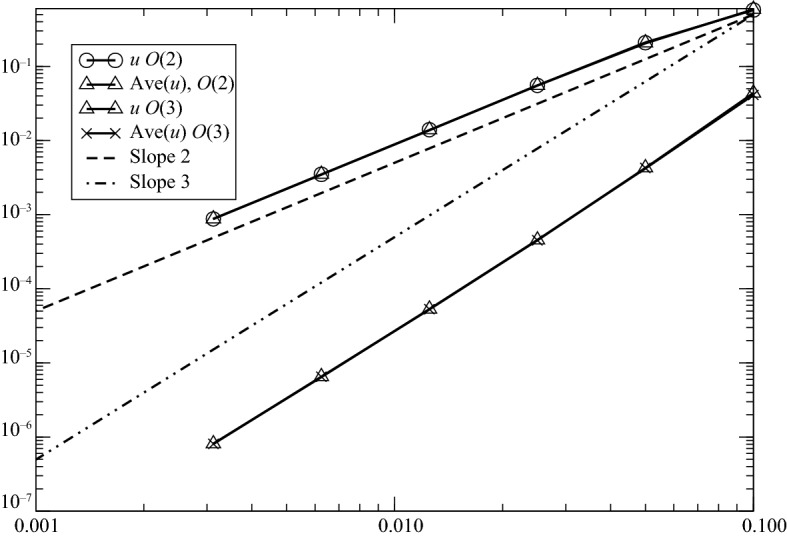
Error plot for *u* and ${\bar{u}}$ for (17) with SSPKR2 and SSPRK3 (CFL = 0.4). Here $f(u)=u$. The second-order results are obtained with SSPRK2 with (17a) and (17b), the third-order results are obtained by (17a) and (17b)

Here, the mesh is uniform, so that $\Delta _{j+1/2}=\Delta$ for any $j\in {\mathbb{Z}}$. It is easy to check the consistency, and in Fig. 1 we show the $L^1$ error on *u* and ${\bar{u}}$ for (17) with SSPKR2 and SSPRK3 (CFL = 0.4) for a convection problem

$$\begin{aligned} \frac{\partial u}{\partial t}+\frac{\partial u}{\partial x}=0 \end{aligned}$$

with periodic boundary conditions and the initial condition $u_0=\cos (2\uppi x)$.

#### **Remark 2**

(Linear stability) In Appendix B, we perform the $L^2$ linear stability and we get, with $\lambda =\frac{\Delta t}{\Delta }$,

- first-order scheme, $\vert \lambda \vert \leqslant 0.92$,
- second-order scheme, $\vert \lambda \vert \leqslant 0.6$,
- third-order scheme, $\vert \lambda \vert \leqslant 0.5$.

**Fig. 2:**
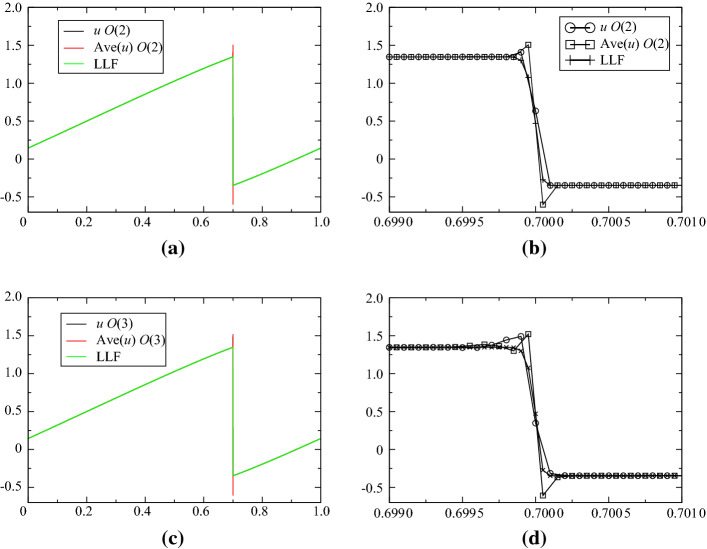
Solution of Burgers with 10 000 points, $t_{{\mathrm{fin}}}=0.4$, $\text{CFL}=0.4$ for the second order (**a**, **b**) [(17a) and (17b) with SSPRK2] and third order (**c**, **d**) [(17a) and (17b) with SSPRK3]. The global solution is represented in **a** and **c**, and a zoom around the discontinuity is shown in **b** and **c**

We also have run this scheme for the Burgers equation, and compared it with a standard finite volume (with local Lax-Friedrichs). The conservative form of the PDE is used for the average, and the non-conservative one for the point values: $J=u$ and $\psi (u)=u$. This is an experimental check of conservation. The initial condition is

$$\begin{aligned} {\mathbf{u}}_0(x)=\sin (2\uppi x)+\frac{1}{2} \end{aligned}$$

on [0, 1], so that there is a moving shock (Fig. 2).

We can see that the agreement is excellent and that the numerical solution behaves as expected.

### Non-linear Stability

As such, the scheme is at most linearly stable, with a CFL condition based on the fine grid. However, in case of discontinuities or the occurrence of gradients that are not resolved by the grid, we have to face oscillations, as usual.

In order to get high-order oscillation free results, a natural option would be to extend the MUSCL approach to the present context. However, it is not very clear how to proceed, so we have relied on the MOOD paradigm [8, 24]. The idea is to work with several schemes ranging from order *p* to 1, with the lowest-order it is able to provide results with positive density and pressure. These schemes are the $S_{j+1/2}(k), k=1, \cdots ,3$ schemes defined above. They are assumed to work for a given CFL range, and the algorithm is as follows: for each Runge-Kutta sub-step, starting from $U^n=\{\bar{\mathbf{u}}_{j+1/2}^n, \bar{\mathbf{v}}_j^n\}_{j\in {\mathbb{Z}}}$, we compute

18 $$\left \{\begin{aligned} \tilde{\bar{\mathbf{u}}}_{j+1/2}^{n+1}&=\bar{\mathbf{u}}_{j+1/2}^n -\lambda _n\big( {{\mathbf{f}}}({\mathbf{u}}_{j+1}^n)-{{\mathbf{f}}}({\mathbf{u}}_j^n)\big), \quad \lambda _n=\frac{\Delta t_n}{\Delta _{j+1/2}},\\ {\tilde{{\mathbf{v}}}}_j^{n+1}&={\tilde{{\mathbf{v}}}}_j^{n} - 2\Delta t_n\overleftarrow{\Phi }^{\mathbf{v}}_{j+1/2},\\ {\tilde{{\mathbf{v}}}}_{j+1}^{n+1}&={\tilde{{\mathbf{v}}}}_{j+1}^{n}-2\Delta t_n\overrightarrow{\Phi }^{\mathbf{v}}_{j+1/2} \end{aligned}\right.$$

by the scheme $S_{j+1/2}(p)$. Then we test the validity of these results in the interval $[x_{j}, x_{j+1}]$ for the density (and possibly the pressure). This is described a little bit later. The variable ${\mathbf{v}}$ is updated as in (18), because at $t_{n+1}$, the true update of ${\mathbf{v}}_j$ is the half sum of ${\tilde{{\mathbf{v}}}}_j^{n+1}$ and ${\tilde{{\mathbf{v}}}}_{j+1}^{n+1}$.

If the test is positive, then we keep the scheme $S_{j+1/2}(p)$ in that interval, else we start again with $S_{j+1/2}(p-1)$, and repeat the procedure unless all the intervals $K_{j+1/2}$ have successfully passed the test. This is described in Algorithm 1 where ${\mathcal{S}}_{j+1/2}$ is the stencil used in $K_{j+1/2}$.

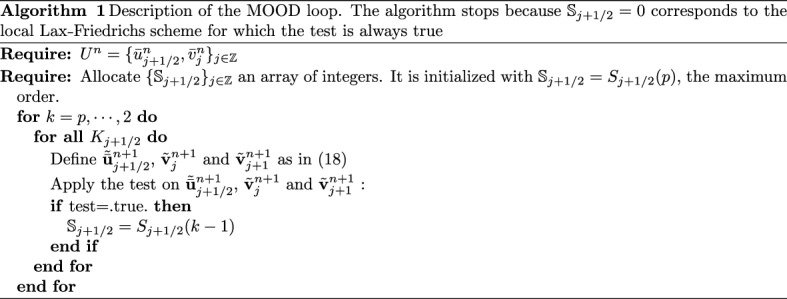



Now, we describe the tests. We do, in the following order, for each element $K_{j+1/2}$, at the iteration $k>0$ of the loop of (i): the tests are performed on variables evaluated from ${\mathbf{u}}$ and ${\mathbf{v}}$. For the scalar case, they are simply the point values at $x_j, x_{j+1/2}$, and $x_{j+1}$. For the Euler equations they are the density, and possibly the pressure.

(i) We check if all the variables are numbers (i.e., not NaN). If not, we state ${\mathbb{S}}_{j+1/2}=S_{j+1/2}(k-1)$.
(ii) (Only for the Euler equations) We check if the density is positive. We can also request to check if the pressure is also positive. If the variable is negative, then we state that ${\mathbb{S}}_{j+1/2}=S_{j+1/2}(k-1)$.
(iii) Then we check if at $t_n$, the solution was not constant in the numerical stencils of the degrees of freedom in $K_{j+1}$, in order to avoid detecting a fake maximum principle. We follow the procedure of [24]. If we observe that the solution was locally constant, then ${\mathbb{S}}_{j+1/2}$ is not modified.
(iv) Then we apply a discrete maximum principle, even for systems though it is not very rigorous. For the variable $\xi$ (in practice the density, and we may request to do the same on the pressure), we compute $\min _{j+1/2}\xi$ (resp. $\max _{j+1/2}\xi$) the minimum (resp. maximum) of the values of $\xi$ on $K_{j+1/2}$, $K_{j-1/2}$, and $K_{j+3/2}$. We say we have a potential maximum if ${\tilde{\xi }}^{n+1}\not \in [\min _{j+1/2}\xi ^n+\varepsilon _{j+1/2}, \max _{j+1/2}\xi ^n-\varepsilon _{j+1/2}]$with $\epsilon _{j+1/2}$ estimated as in [8]. Then we get the followings.
  If ${\tilde{\xi}}^{n+1}\in [\min _{j+1/2}\xi ^n+\varepsilon _{j+1/2}, \max _{j+1/2}\xi ^n-\varepsilon _{j+1/2}]$, then ${\mathbb{S}}_{j+1/2}$ is not modified.
  Else we use the following procedure introduced in [24]. In each $K_{l+1/2}$, we can evaluate a quadratic polynomial $p_{l+1/2}$ that interpolates $\xi$. Note that its derivative is linear in $\xi$. We compute $$\begin{aligned} p'_{j-1/2}(x_j), p'_{j+3/2}(x_{j+1}), p'_{j+1/2}(x_j) \quad \text{and}\quad p'_{j+1/2}(x_{j+1}). \end{aligned}$$
    If $$\begin{aligned}&p'_{j+1/2}(x_j) \in [\min (p'_{j-1/2}(x_j),p'_{j+3/2}(x_{j+1})]\\ &\quad \text{and}\quad p'_{j+1/2}(x_{j+1}) \in [\min (p'_{j-1/2}(x_j),p'_{j+3/2}(x_{j+1})], \end{aligned}$$we say it is a true regular extrema and ${\mathbb{S}}_{j+1/2}$ will not be modified.
    Else the extrema is declared not to be regular, and ${\mathbb{S}}_{j+1/2}=S_{j+1/2}(k-1).$

As a first application, to show that the oscillations are well controlled without sacrificing the accuracy, we consider the advection problem (with constant speed unity) on [0, 1], periodic boundary conditions with the initial condition:

$$\begin{aligned} u_0(x)= \left\{ \begin{array}{ll} 0&{}\text{ if } y\in [-1,-0.8[,\\ \frac{1}{6}\big(G(y, \beta , z-\delta )+G(y,\beta ,z+\delta )+4G(y,\beta ,z)\big) &{} \text{ if }y\in [-0.8,-0.6],\\ 1&{} \text{ if } y\in [-0.4,-0.2]\\ 1-\vert 10y-1\vert &{} \text{ if }y\in [0,0.2],\\ \frac{1}{6}\big(F(y, \beta , z-\delta )+G(y,\beta ,z+\delta )+4F(y,\beta ,z)\big) &{} \text{ else.} \end{array} \right. \text{ with } y=2x-1, \end{aligned}$$

Here $$a=0.5$$, $$z=-0.7$$, $$\delta =0.005$$, $$\alpha =10$$,

$$\begin{aligned} \beta =\frac{\log 2}{36\delta ^2} \end{aligned}$$

and

$$\begin{aligned} G(t,\beta ,z)=\exp \big( -\beta (t-z)^2\big), \quad F(t,a,\alpha )=\sqrt{\max \big(0, 1-\alpha (t-a)^2\big)}. \end{aligned}$$

**Fig. 3:**
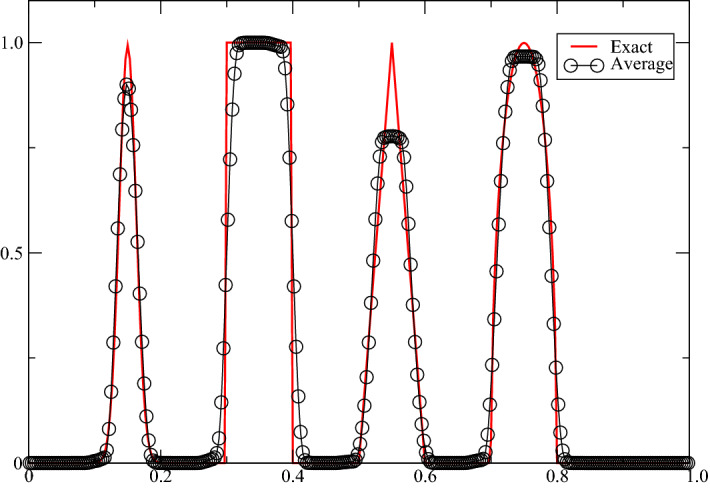
Shu-Jiang problem, CFL = 0.4, third-order scheme with MOOD, 300 points, periodic conditions, 10 periods. The point values and cell average are almost undistinguishable

Using the MOOD procedure with the third-order scheme, the results obtained for 300 points for $T=10$ are displayed in Fig. 3. They look very reasonable.

## Numerical Results for the Euler Equations

In this section, we show the flexibility of the approach, where conservation is recovered only by (17a), and so lots of flexibility is possible with the relations on the ${\mathbf{u}}_i$. To illustrate this, we consider the Euler equations. We will consider the conservative formulation (1) for the average value, so ${\mathbf{u}}=(\rho , \rho u, E)^{\mathrm{T}}$ and either the form (2), i.e., ${\mathbf{v}}=(\rho , u, p)$ or the form (3) with ${\mathbf{v}}=(p,u,s)^{\mathrm{T}}$.

### Sod Test Case

The Sod case is defined for [0, 1], the initial condition is

$$\begin{aligned} (\rho ,u,p)^{\mathrm{T}}=\left\{ \begin{array}{ll} (1,0,1)^{\mathrm{T}} &{} \quad \text{for } x<0.5,\\ (0.125,0,0.1)^{\mathrm{T}} &{}\quad \text{else}. \end{array}\right. \end{aligned}$$

**Fig. 4:**
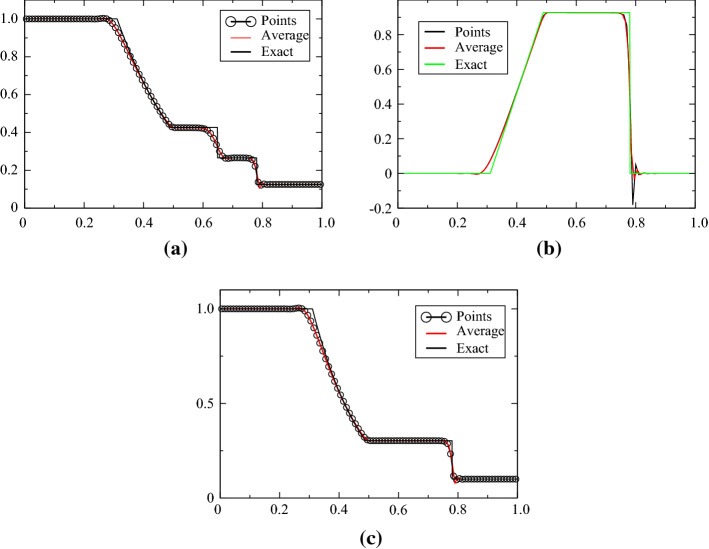
One hundred grid points, and the second-order SSPRK2 scheme with CFL = 0.1. **a** density, **b** velocity, **c** pressure

**Fig. 5:**
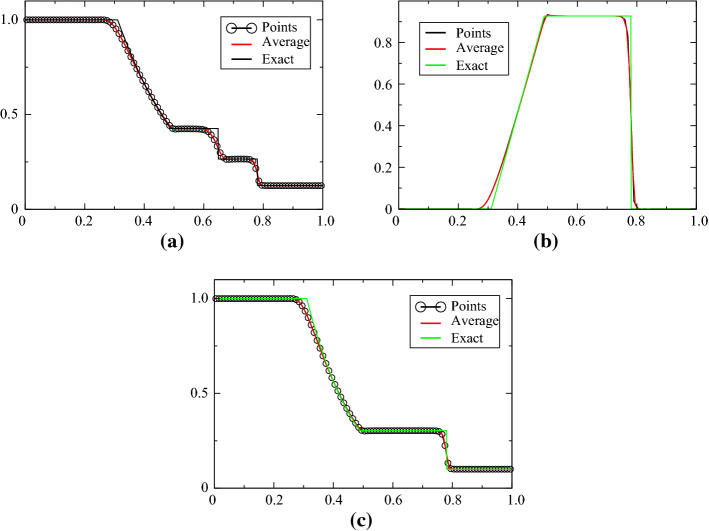
One hundred grid points, and the second-order SSPRK2 scheme with CFL = 0.1. **a** density, **b** velocity, **c** pressure. MOOD test made on $\rho$ and *p*

**Fig. 6:**
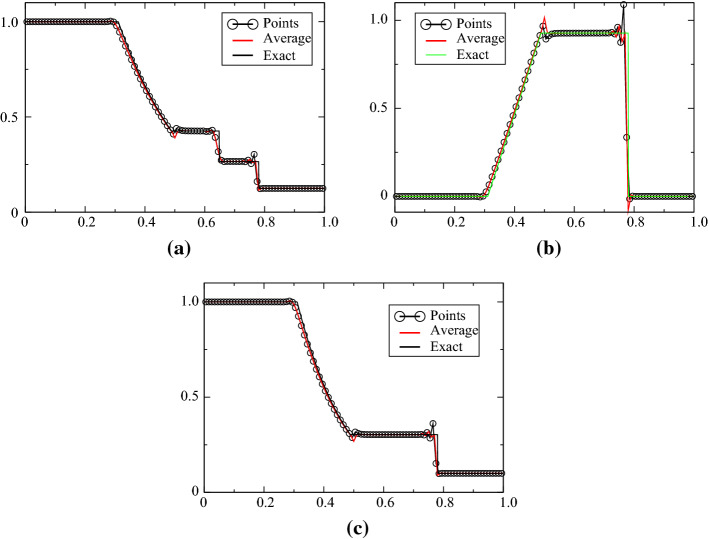
One hundred grid points, and the third-order SSPRK3 scheme with CFL = 0.1. **a** density, **b** velocity, **c** pressure

**Fig. 7:**
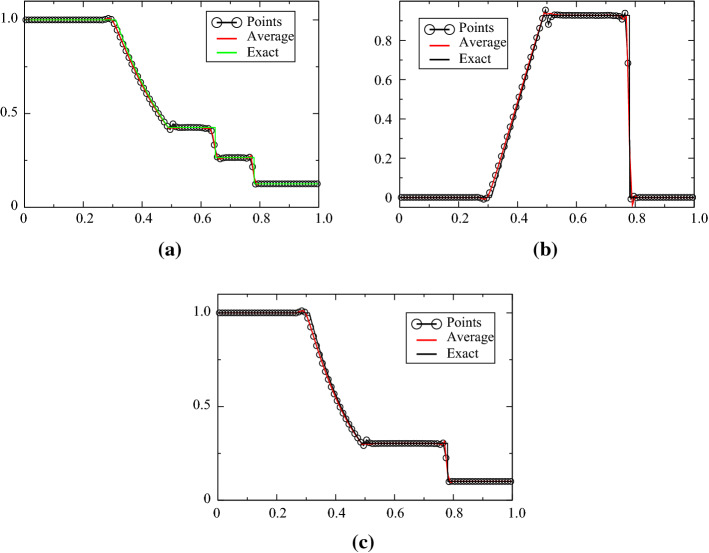
One hundred grid points, and the third-order SSPRK3 scheme with CFL = 0.1. **a** density, **b** velocity, **c** pressure. Mood test made on $\rho$ and *p*

**Fig. 8:**
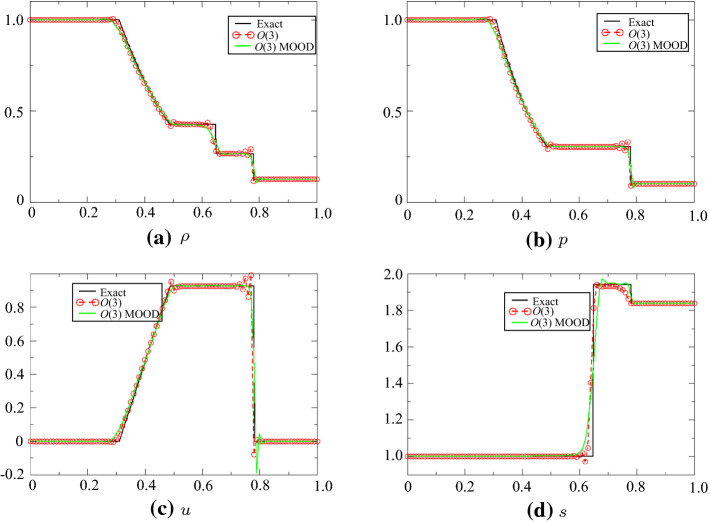
Solution with the variables (*s*, *u*, *p*) for 100 points, comparison with the exact solution, third order in time/space with MOOD and non MOOD. MOOD is done on $\rho$ and *p*. CFL = 0.2

**Fig. 9:**
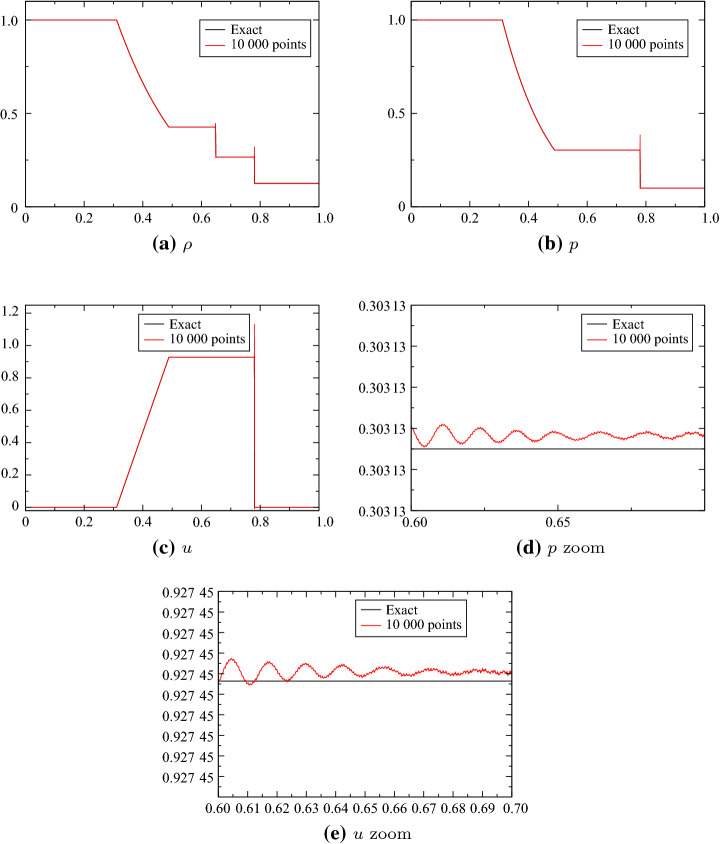
Solution with the variables (*s*, *u*, *p*) for 10 000 points, comparison with the exact solution. CFL = 0.1, no MOOD. The zoomed figures are for $x\in [0.6,0.7]$ and the ticks are for $10^{-7}$. We plot *u* and *p* across the contact

The final time is $T=0.16$. The problem is solved with (1) and (2) and displayed in Figs. 4, 5, 6 and 7, while the solution obtained with the combination (1)–(3) is shown in Figs. 8 and 9. When the MOOD procedure is on, it is applied with $\rho$ and *p* and all the tests are performed. The exact solution is also shown every time. Different orders in time/space are tested. The results are good, even though the MOOD procedure is not perfect. The use of the combination (1)–(3) seems more challenging, we have performed a convergence study (with 10 000 points). This is shown in Fig. 9, and a zoom around the contact discontinuity is also shown.

We can observe a numerical convergence to the exact one in all cases. In Appendix C, we show some results on irregular meshes, with the same conclusions.

### A Smooth Case

We consider a fluid with $\gamma =3$: the characteristics are straight lines. The initial condition is inspired from Toro: in $[-1,1]$,

19 $$\left \{\begin{aligned} \rho _0(x)&=1+\alpha \sin (2\uppi x),\\ u_0(x)&=0,\\ p_0(x)&=\rho _0(x)^\gamma . \end{aligned}\right.$$

The classical case is for $\alpha =0.999{\;} 995$ where the vacuum is almost reached. Here, since we do not want to test the robustness of the method, we take $\alpha =\frac{3}{4}$. The final time is set to $T=0.1$.

The exact density and velocity in this case can be obtained by the method of characteristics and are explicitly given by

$$\begin{aligned} \rho (x,t) = \frac{1}{2}\big( \rho _0(x_1) + \rho _0(x_2)\big), \quad u(x,t) = \sqrt{3}\big(\rho (x,t)-\rho _0(x_1) \big), \end{aligned}$$

where for each coordinate *x* and time *t* the values $x_1$ and $x_2$ are solutions of the non-linear equations

$$\begin{aligned}&x + \sqrt{3}\rho _0(x_1) t - x_1 = 0, \\ &x - \sqrt{3}\rho _0(x_2) t - x_2 = 0. \end{aligned}$$

**Fig. 10:**
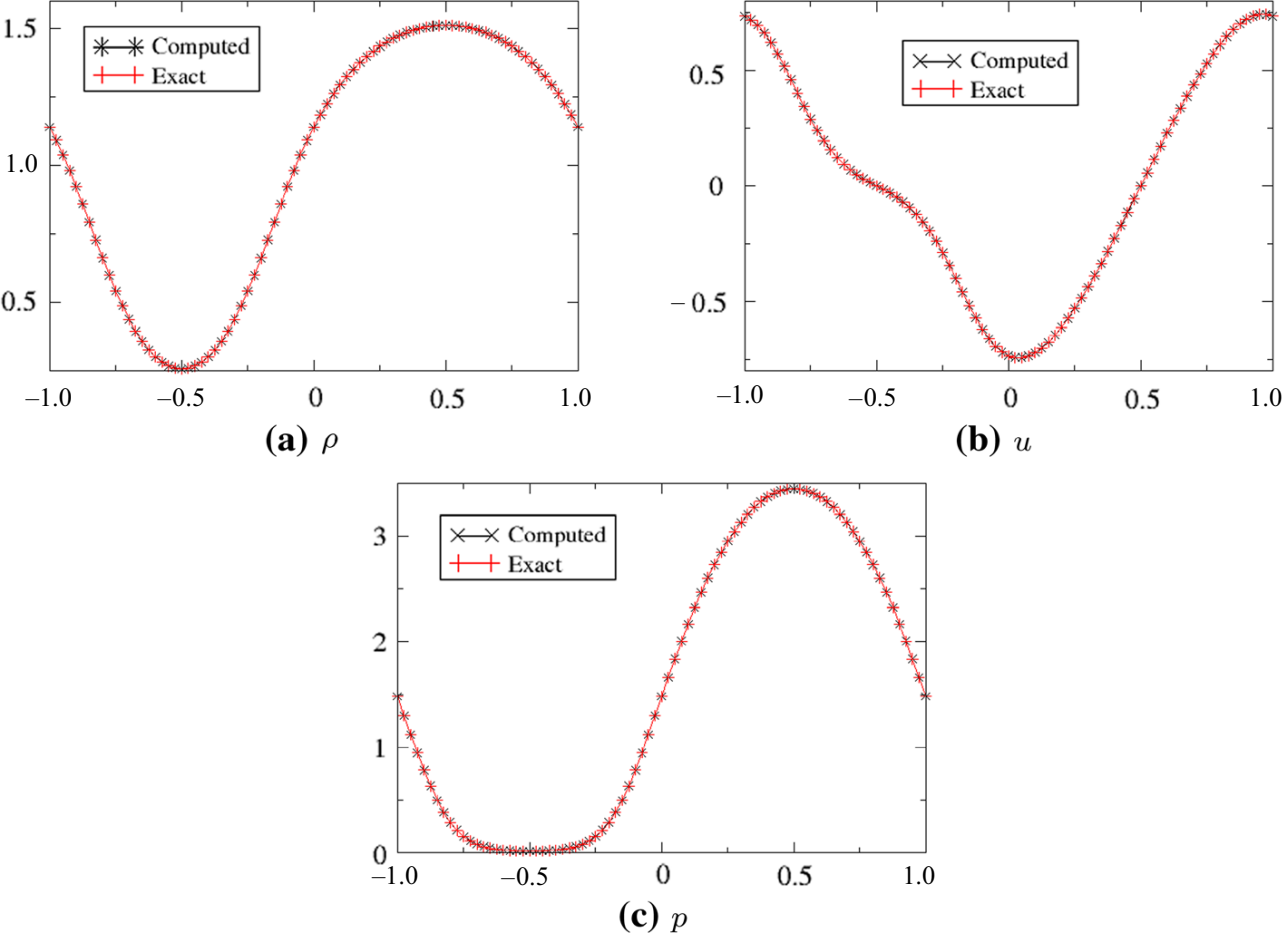
Solutions (numerical and exact) for the conditions (19). The number of grid points is set to 80, with periodic boundary conditions

An example of the numerical solution, superimposed with the exact one, is shown in Fig. 10. It is obtained with the third-order (time and space) scheme, and here we have used the model $(\rho ,u,p)$. The CFL number is set to 0.2.

The errors are shown in Table 1.

**Table 1:** $L^1$, $L^2$ and $L^\infty$ errors for the initial conditions (19) with the third-order scheme

$h=1/N$	$L^1$		$L^2$		$L^\infty$	
20	$2.136\times 10^{-4}$	−	$2.968\times 10^{-4}$	−	$6.596\times 10^{-4}$	−
40	$1.912\times 10^{-5}$	$-3.48$	$2.702\times 10^{-5}$	$-3.45$	$5.750\times 10^{-5}$	$-3.52$
80	$1.398\times 10^{-6}$	$-3.77$	$2.138\times 10^{-6}$	$-3.65$	$4.673\times 10^{-6}$	$-3.62$
160	$1.934\times 10^{-7}$	$-2.85$	$2.595\times 10^{-7}$	$-3.04$	$5.753\times 10^{-7}$	$-3.02$
320	$3.641\times 10^{-8}$	$-2.40$	$5.523\times 10^{-8}$	$-2.23$	$1.276\times 10^{-7}$	$-2.17$

The errors, computed in $[-1,1]$ are in reasonable agreement with the $-3$ expected slopes. We also have done the same test with the non-linear stabilisation procedure described in Sect. 3.2. Exactly the same errors are obtained: the order reduction test is never activated.

### Shu-Osher Case

**Fig. 11:**
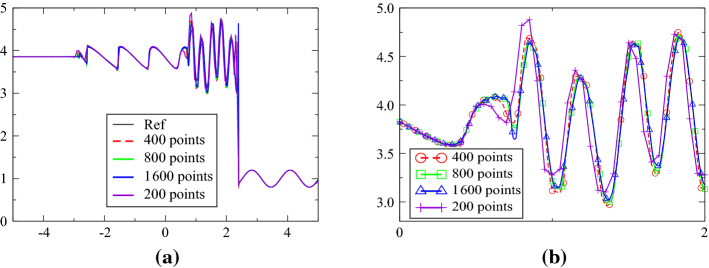
**a** Solution of the Shu-Osher problem, **b** zoom of the solution around the shock

The initial conditions are

$$\begin{aligned} (\rho , u, p)=\left\{ \begin{array}{ll} (3.857 \,143, 2.629 \,369, 10.333{\;}333{\;}3) &{}\text{if } x<-4,\\ (1+0.2\sin (5x), 0, 1) &{}\text{else} \end{array}\right. \end{aligned}$$

on the domain $[-5,5]$ until $T=1.8$. We have used the combination (1) and (2), since another one seems less robust. The density is compared to a reference solution (obtained with a standard finite volume scheme with 20 000 points, and the solution obtained with the third-order scheme with $\text{CFL}=0.3$ and 200, 400, 800 and 1 600 points. The MOOD procedure uses the first-order upwind scheme if a PAD, a NaN or a DMP is detected, the other cases use the third-order scheme. The solutions are displayed in Fig. 11. With little resolution, the results are very close to the reference one.

For Fig. 11, the second-order scheme is used as a rescue scheme.

### Le Blanc Case

**Fig. 12:**
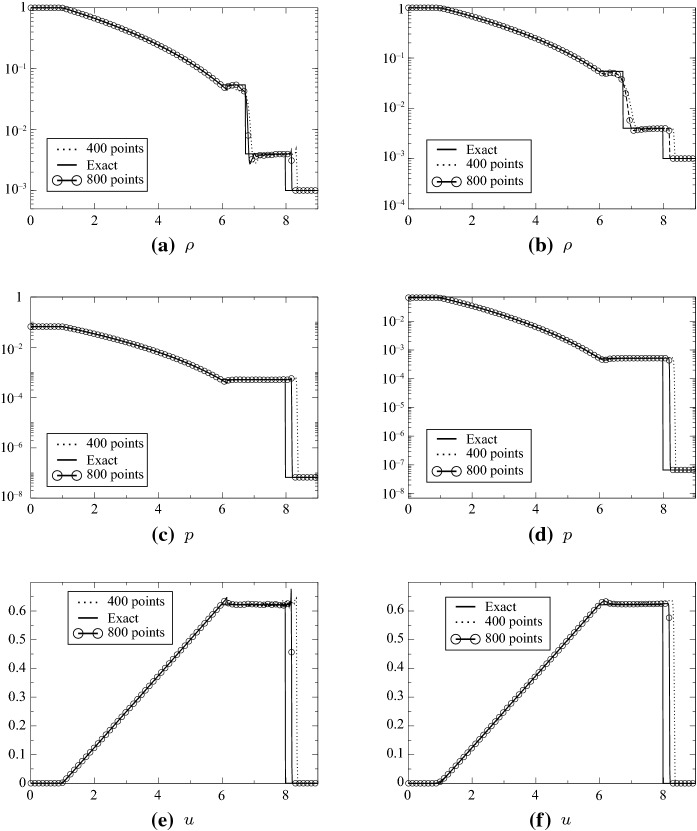
Le Blanc test case, $\text{CFL}=0.1$, from 400 to 800 points. Left column: MOOD test on $\rho$ and *p*, second order; right column: MOOD test on $\rho$ and *p*, third order

**Fig. 13:**
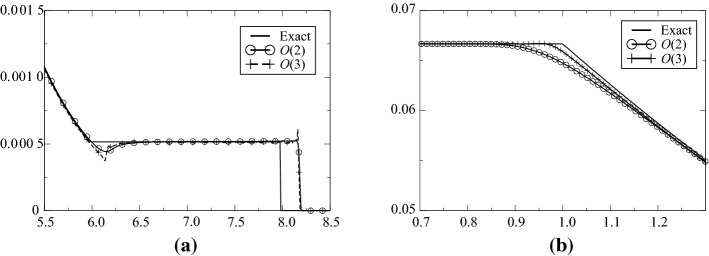
Le Blanc test case, zooms, comparison on the pressure between second order and third order with 400 points

The initial conditions are

$$\begin{aligned} (\rho ,u,e)=\left\{ \begin{array}{ll} (1,0,0.1) &{} \quad \text{if } x\in [-3,3],\\ (0.001,0.10^{-7} ) &{} \quad \text{if } x\in [3,6], \end{array} \right. \end{aligned}$$

where $e=(\gamma -1) p$ and $\gamma =\frac{5}{3}$. The final time is $t=6$. This is a very strong shock tube and we use the combination (1) and (2). It is not possible to run higher than first order without the MOOD procedure. The second- and third-order results are shown in Fig. 12, and zooms around the shocks and the fan are showed in Fig. 13.

**Fig. 14:**
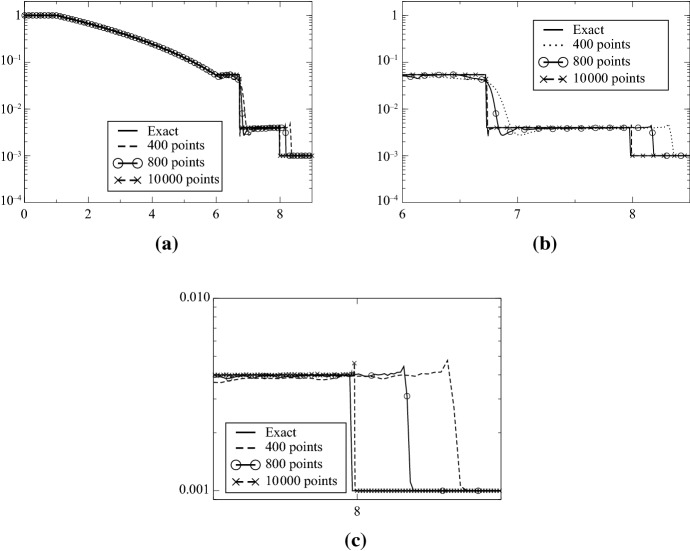
Convergence study on the density for the Le Blanc test case

At time $t=6$, the shock wave should be at $x=8$: in addition to the extreme conditions, it is generally difficult to get a correct position of the shock wave; this is why a convergence study is shown in Fig. 14. It is performed with 400, 800, 10 000 grid points, and the third-order SSPRK3 scheme with CFL = 0.1. It is compared to the exact solution, and the results are good, see for example [22] for a comparison with other methods, or [21] for a comparison with Lagrangian methods.

## Conclusion

This study is preliminary and should be seen as a proof of concept. We show how to combine, without any test, several formulations of the same problem, one in the conservative form and the others in the non-conservative form, to compute the solution of hyperbolic systems. The emphasis is mostly put on the Euler equations.

We explain why the formulation leads to a method that satisfies a Lax-Wendroff-like theorem. We also propose a way to provide the non-linearity stability, and this method works well but is not yet completely satisfactory.

Besides the theoretical results, we also show numerically that we get the convergence to the correct weak solution. This is done on standard benchmark and it is very challenging.

We intend to extend the method to several space dimensions and improve the limiting strategy. Different systems, such as the shallow water system, will also be considered.

## Figures and Tables

**Fig. A1:**
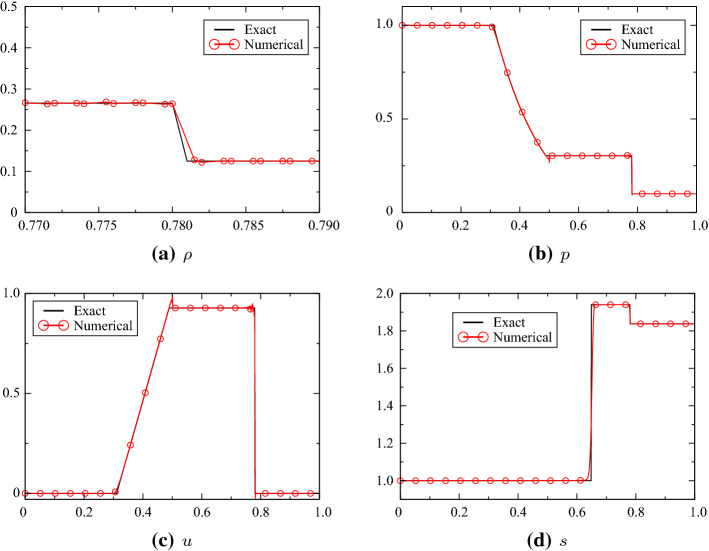
Plot of the density, pressure, velocity and the pressure. This is obtained with the “third order” with MOOD test on the density and the pressure

**Fig. A2:**
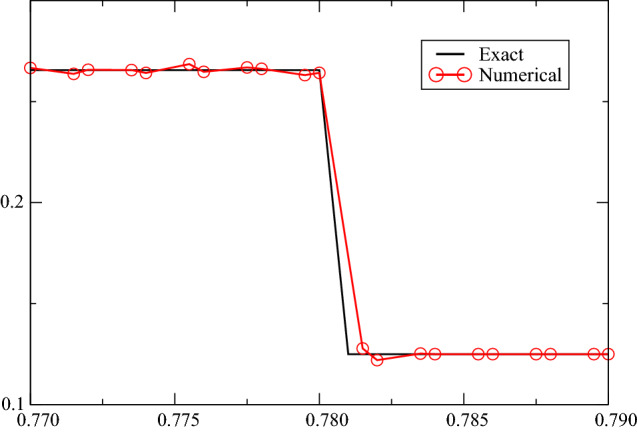
Zoom of the density around the shock wave

## Appendix A Proof of Proposition 1

We show Proposition 1 in the scalar case, and the system case is identical.

We start by some notations: ${\mathbb{R}}$ is subdivided into intervals $K_{j+1/2}=[x_j,x_{j+1}]$ with $x_j<x_{j+1}$, and *h* will be the maximum of the length of $K_{j+1/2}$. On each interval, from the point values $u_i$ and $u_{i+1}$, as well as the average $\bar{u}_{j+1/2}$, we can construct a quadratic polynomial. From this and as above, we can construct a globally continuous piecewise quadratic function and it is denoted by $R_{u _\Delta }$.

Let $T>0$ and a time discretisation $0<t_1<\cdots<t_n<\cdots <t_N\leqslant T$ of [0, *T*]. We define $\Delta t_n=t_{n+1}-t_n$ and $\Delta t=\max _n \Delta t_n$. We are given the sequences $\{u_j^p\}_{j\in {\mathbb{Z}}}^{p=0,\cdots, N}$ and $\{{\bar{u}}_{j+1/2}^n\}_{j\in {\mathbb{Z}}}^{p=0, \cdots , N}$. We can define a function $u_{\Delta }$ by

$$\begin{aligned} \text{if }(x,t)\in [x_j,x_{j+1}]\times [t_{n}, t_{n+1}[,\quad \text{then } u _\Delta (x,t)=R_{u^n_\Delta }(x). \end{aligned}$$

The set of these functions is denoted by $X _{\Delta }$ and is equipped with the $L^\infty$ and $L^2$ norms.

We have the following lemma.

### **Lemma A1**

*Let* $T>0,$ $\{t_n\}_{n=0, \cdots , N}$ *be an increasing subdivision of* [0, *T*],  [*a*, *b*] *a compact of* ${\mathbb{R}}.$ *Let* $(u _\Delta )_{h}$ *be a sequence of functions of* $X _\Delta$ *defined on* ${\mathbb{R}}\times {\mathbb{R}}^+.$ *We assume that there exists* $C\in {\mathbb{R}}$ *independent of* $\Delta$ *and* $\Delta t,$ *and* ${\mathbf{u}}\in L^2_{{\mathrm{loc}}}([a,b]\times [0,T])$ *such that*

$$\begin{aligned} \sup \limits _\Delta \sup \limits _{x,t}\vert u _\Delta (x,t)\vert \leqslant C\quad \text{and}\quad \lim \limits _{\Delta , \Delta t\rightarrow 0}\vert u _\Delta -u\vert _{L^2([a,b]\times [0,T])}=0. \end{aligned}$$

*Then*

A1 $$\begin{aligned} \lim \limits _{\Delta , \Delta t\rightarrow 0}\sum _{n=0}^N\Delta t_n \bigg[\sum _{j\in {\mathbb{Z}}}\Delta _{j+1/2}\bigg( \vert u_{j}^n-\bar{u} _{j+1/2}^{n}\vert + \vert u_{j+1}^n-\bar{u} _{j+1/2}^{n}\vert +\vert u_{j}^n-u_{j+1}^n\vert \bigg)\bigg] =0. \end{aligned}$$

### **Proof**

First, because the vector space of polynomials of degree 3 on $[x_j,x_{j+1}]$ is finite-dimensional and with a dimension independent of *j*, there exist $C_1$ and $C_2$ such that

$$\begin{aligned} &C_1 \Delta _{j+1/2}\bigg( \vert u_{ j}^n-\bar{u} _{j+1/2}^{n}\vert + \vert u_{ j+1}^n-\bar{u} _{j+1/2}^{n}\vert \bigg)\leqslant \int _{x_j}^{x_{j+1}} \vert u _\Delta (x,t_n)-{{\bar{u}}} _{j+1/2}^n\vert {\mathrm{d}}x\\ &\qquad \qquad \qquad \qquad \qquad \qquad \qquad  \qquad \qquad \quad \leqslant C_2 \Delta _{j+1/2}\bigg( \vert u_{ j}^n-\bar{u} _{j+1/2}^{n}\vert + \vert u_{j+1}^n-\bar{u} _{j+1/2}^{n}\vert \bigg), \end{aligned}$$

so that

$$\begin{aligned} \sum _{n=0}^K \Delta t_n \sum \limits _{j, K_{j+1/2}\subset [a,b]} \Delta _{j+1/2}\bigg( \vert u_{ j}^n-\bar{u} _{j+1/2}^{n}\vert + \vert u_{ j+1}^n-\bar{u} _{j+1/2}^{n}\vert \bigg)\leqslant C_1^{-1} \int _0^{\mathrm{T}}\int _a^b \vert u _\Delta -{\bar{u}} _\Delta \vert {\mathrm{d}}x, \end{aligned}$$

where for simplicity we have introduced ${\bar{u}} _\Delta$ the function defined by

$$\begin{aligned} \text{if }(x,t)\in [x_j,x_{j+1}[\times [t_n, t_{n+1}[, {\bar{u}}_\Delta (x,t)={{\bar{u}}}_{j+1/2}^{n}. \end{aligned}$$

Then we rely on classical arguments of functional analysis: since $(u _\Delta )$ is bounded and $L^\infty ([a,b]\times [0,T]) \subset L^1([a,b]\times [0,T])$, there exists $u'\in L^\infty ([a,b]\times [0,T]$ such that $u _\Delta \rightarrow u'$ in the weak-$\star$ topology. Similarly, there exists ${\bar{u}}\in L^\infty ([a,b]\times [0,T])$ such that ${{\bar{u}}} _\Delta \rightarrow {\bar{u}}$ for the weak-$\star$ topology.

Since $u _\Delta \rightarrow u$ in $L^2_{{\mathrm{loc}}}$, we have $u'=u$ because $[a,b]\times [0,T]$ is bounded and $C_0^\infty ([a,b]\times [0,T])$ is dense in $L^1([a,b]\times [0,T])$. Let us show that ${\bar{u}}=u$. let $\varphi \in C_0^\infty ({\mathbb{R}}\times {\mathbb{R}}^+)$. We have, setting

$$\begin{aligned}&\bar{\varphi }_{j+1/2}^n=\frac{1}{\Delta _{j+1/2}\Delta t_n}\int _{t_n}^{t_{n+1}} \int _{x_j}^{x_{j+1}} \varphi (x,t) {\mathrm{d}}x{\mathrm{d}}t,\\ &\begin{aligned} \int _0^{T} \int _a^b \big( {\bar{u}} _\Delta -u _\Delta \big) \varphi {\mathrm{d}}x {\mathrm{d}}t&=\sum _n \sum \limits _{a\leqslant x_j<x_{j+1}\leqslant b} \int _{t_n}^{t_{n+1}}\int _{x_j}^{x_{j+1}} \big( {\bar{u}} _\Delta -u_\Delta \big) \varphi  {\mathrm{d}}x {\mathrm{d}}t\\ &=\sum _n \sum \limits _{a\leqslant x_j<x_{j+1}\leqslant b}\bigg( \int _{t_n}^{t_{n+1}}\int _{x_j}^{x_{j+1}} \big( {\bar{u}} _\Delta -u_\Delta \big) \varphi  {\mathrm{d}}x {\mathrm{d}}t\\ &\qquad -\int _{t_n}^{t_{n+1}}\int _{x_j}^{x_{j+1}} \big( {\bar{u}} _\Delta -u_\Delta \big) \bar{\varphi }_{j+1/2}^n {\mathrm{d}}x {\mathrm{d}}t\bigg)\\ &=\sum _n \sum \limits _{a\leqslant x_j<x_{j+1}\leqslant b}\int _{t_n}^{t_{n+1}}\int _{x_j}^{x_{j+1}}\big( {\bar{u}} _\Delta -u_\Delta \big)\big( \varphi -\bar{\varphi }_{j+1/2}^n\big)  {\mathrm{d}}x {\mathrm{d}}t \end{aligned} \end{aligned}$$

and using the fact that for any $[x_j, x_{j+1}]\times [t_n, t_{n+1}]$, we have $\int _{t_n}^{t_{n+1}}\int _{x_j}^{x_{j+1}} \big( {\bar{u}} _\Delta -u_\Delta \big) {\mathrm{d}}x {\mathrm{d}}t=0$.

Since $\varphi \in C_0^\infty ({\mathbb{R}}\times {\mathbb{R}}^+)$, there exists *C* that depends only on $\Vert \frac{{\mathrm{d}}\varphi }{{\mathrm{d}}x}\Vert _{L^\infty ({\mathbb{R}}\times {\mathbb{R}}+)}$ such that

$$\begin{aligned}&\bigg\vert \int _{x_j}^{x_{j+1}} \big({\bar{u}} _\Delta -u_\Delta \big)\big( \varphi -\bar{\varphi }\big)  {\mathrm{d}}x {\mathrm{d}}t\bigg\vert \leqslant \Delta t \Delta _{j+1/2}\; \Delta \max \limits _{j\in {\mathbb{Z}}, n\leqslant N}\big( \vert u_j^n\vert , \vert {{\bar{u}}}_{j+1/2}^n\vert \big)\\ &\quad \quad \quad \quad \quad \quad \quad \quad \quad \quad \qquad \qquad \leqslant \Delta t \Delta ^2 \max \limits _{j\in {\mathbb{Z}}, n\leqslant N}\big( \vert u_j^n\vert , \vert {{\bar{u}}}_{j+1/2}^n\vert \big), \end{aligned}$$

and then

$$\begin{aligned} \bigg\vert \int _0^{T} \int _a^b \big({{\bar{u}}} _\Delta -u _\Delta \big) \varphi \; {\mathrm{d}}x {\mathrm{d}}t\bigg\vert \leqslant C \Delta, \end{aligned}$$

and passing to the limit, ${\bar{u}}=u'$. Since a subsequence of $u _\Delta$ converges to *u* in $L^2$, we have ${\bar{u}}=u'=u$.

The same method shows that $(u _\Delta ^2)$ and $({{\bar{u}}} _\Delta ^2)$ have the same weak-$\star$ limit. Let us show it is $u^2$. Since $C_0^\infty ([a,b]\times [0,T]$ is dense in $L^1([a,b]\times [0,T]$) and $u _\Delta ^2$ is bounded independently of $\Delta$ and $\Delta t$, we can choose functions $\phi$ in $C_0^\infty ([a,b]\times [0,T])$. This test function is bounded in $[a,b]\times [0,T]$ and we have at least for a subsequence,

$$\begin{aligned} \int _{a}^b\int _{0}^{T} \vert u-u _\Delta \vert ^2  {\mathrm{d}}x {\mathrm{d}}t \rightarrow 0, \end{aligned}$$

and then

$$\begin{aligned} \int _a^b\int _{0}^{T} u _\Delta ^2 {\mathrm{d}}x {\mathrm{d}}t-2\int _a^b\int _{0}^{T} u _\Delta \, u {\mathrm{d}}x {\mathrm{d}}t+ \int _a^b\int _{0}^{T} u^2 {\mathrm{d}}x {\mathrm{d}}t\rightarrow 0. \end{aligned}$$

By the Cauchy-Schwarz inequality, $u\phi \in L^1([a,b]\times [0,T])$: the second term tends towards

$$\begin{aligned} \int _a^b\int _{0}^{T} u^2 {\mathrm{d}}x {\mathrm{d}}t, \end{aligned}$$

so that

$$\begin{aligned} \int _a^b\int _{0}^{T} u _\Delta ^2 {\mathrm{d}}x {\mathrm{d}}t- \int _a^b\int _{0}^{T} u^2 {\mathrm{d}}x {\mathrm{d}}t\rightarrow 0, \end{aligned}$$

and $u _\Delta ^2\rightarrow u^2$ in $L^\infty$ weak-$\star$.

Last, again by the same argument for $\phi =1$, since $u _\Delta ^2\rightarrow u^2$ in $L^\infty$ weak-$\star$, we get

$$\begin{aligned} \int _a^b\int _{0}^{T}\vert {\bar{u}} _\Delta -u\vert ^2  {\mathrm{d}}x {\mathrm{d}}t\rightarrow 0, \end{aligned}$$

and finally

$$\begin{aligned} \int _a^b\int _{0}^{T}\vert {\bar{u}} _\Delta -u _\Delta \vert ^2  {\mathrm{d}}x {\mathrm{d}}t\rightarrow 0. \end{aligned}$$

Since $[a,b]\times [0,T]$ is bounded and $L^1([a,b]\times [0,T])\subset L^2([a,b]\times [0,T])$, we obtain

$$\begin{aligned} \lim \limits _{ \Delta , \Delta t\rightarrow 0}\sum _{n=0}^N\Delta t_n \bigg[\sum _{j\in {\mathbb{Z}}}\Delta _{j+1/2} ( \vert u_{ j}^n-\bar{u} _{j+1/2}^{n}\vert + \vert u_{j+1}^n-\bar{u} _{j+1/2}^{n}\vert  )\bigg] =0. \end{aligned}$$

From this we get (20) because

$$\begin{aligned} \vert u_{ j}^n-u_{ j+1}^n\vert \leqslant \vert u_{ j}^n-\bar{u} _{j+1/2}^{n}\vert +\vert u_{ j+1}^n-\bar{u} _{j+1/2}^{n}\vert . \end{aligned}$$

Then we can proof Proposition 1. We proceed the proof in several lemmas.

### **Lemma A2**

*Under the conditions of Proposition* 1, *for any* $\varphi \in C_0^\infty ({\mathbb{R}}\times {\mathbb{R}}^+)$ *we have*

$$\begin{aligned}&\lim \limits _{\Delta t\rightarrow 0, \,\Delta \rightarrow 0} \sum \limits _{n=0}^\infty \sum \limits _{[x_j, \,x_{j+1}],\,\nonumber j\in {\mathbb{Z}}}\frac{\Delta _{j+1/2}}{6} ( \varphi _{j+1}^n({\mathbf{u}}_{j+1}^{n+1}-{\mathbf{u}}_{j+1}^n)+4\varphi _{j+1/2}^n ({\mathbf{u}}_{j+1/2}^{n+1}-{\mathbf{u}}_{j+1/2}^n)\\ &\qquad +\varphi _{j}^n({\mathbf{u}}_{j}^{n+1}-{\mathbf{u}}_j^n) )\\ &\quad = -\int _{{\mathbb{R}}\times {\mathbb{R}}^+} \frac{\partial \varphi }{\partial t} u  {\mathrm{d}}x {\mathrm{d}}t+\int _{\mathbb{R}}\varphi (x,0) u_0 {\mathrm{d}}x {\mathrm{d}}t. \end{aligned}$$

### **Proof**

This is a simple adaptation of the classical proof, see for example [14]. We have, using that

$$\begin{aligned} \delta u_{j+1/2}=\frac{3}{2}\overline{\delta u}_{j+1/2}- \frac{\delta u_j+\delta u_{j+1}}{4} \end{aligned}$$

and the compactness of the support of $\varphi$,

$$\begin{aligned} &\sum \limits _{n=0}^\infty \sum \limits _{[x_j, \,x_{j+1}], \,j\in {\mathbb{Z}}}\frac{\Delta _{j+1/2}}{6} ( \varphi _{j+1}^n({\mathbf{u}}_{j+1}^{n+1}-{\mathbf{u}}_{j+1}^n)+4\varphi _{j+1/2}^n ({\mathbf{u}}_{j+1/2}^{n+1}-{\mathbf{u}}_{j+1/2}^n)\\ & + \varphi _{j}^n({\mathbf{u}}_{j}^{n+1}-{\mathbf{u}}_j^n) )\\ & = \underbrace{\sum \limits _{n=0}^\infty \sum \limits _{[x_j, x_{j+1}], j\in {\mathbb{Z}}}\Delta _{j+1/2} \varphi _{j+1/2}\overline{\delta u}_{j+1/2}}_{(\text I)} \\ & + \underbrace{\sum \limits _{n=0}^\infty \sum \limits _{j\in {\mathbb{Z}}}\bigg( \frac{\Delta _{j+1/2}}{6} \big( \varphi _{j}-\varphi _{j+1/2}\big) +\frac{\Delta _{j-1/2}}{6}\big( \varphi _{j}-\varphi _{j-1/2}\big)\bigg)\delta u_j }_{(\text {II})} ,\end{aligned}$$

where $\overline{\delta {\mathbf{u}}}_{j+1/2}=\bar{\mathbf{u}}_{j+1/2}^{n+1}-\bar{\mathbf{u}}_{j+1/2}^{n}$ and $\delta {\mathbf{u}}_{j} ={\mathbf{u}}_j^{n+1}-{\mathbf{u}}_j^n$. The first part, (I), is the classical term, and under the condition of the lemma, converges to

$$\begin{aligned} -\int _{{\mathbb{R}}\times {\mathbb{R}}^+} \frac{\partial \varphi }{\partial t} u  {\mathrm{d}}x {\mathrm{d}}t+\int _{\mathbb{R}}\varphi (x,0) u_0 {\mathrm{d}}x {\mathrm{d}}t. \end{aligned}$$

Since $\varphi \in C_0^\infty ({\mathbb{R}}\times {\mathbb{R}}^+)$, there exists *C* that depends only on the $L^\infty$ norm of the first derivative of $\varphi$ such that the term (II) can be bounded by

$$\begin{aligned}&\bigg\vert \sum \limits _{n=0}^N\Delta t_n \sum \limits _{j\in {\mathbb{Z}}}\bigg( \frac{\Delta _{j+1/2}}{6} \big( \varphi _{j}-\varphi _{j+1/2}\big) +\frac{\Delta _{j-1/2}}{6}\big( \varphi _{j}-\varphi _{j-1/2}\big)\bigg)\delta u_j \bigg\vert \\ & \leqslant C \sum \limits _{n=0}^N \Delta t\Delta \sum \limits _{j\in {\mathbb{Z}}}\Delta _{j+1/2} \vert \delta u_j\vert \\ & \leqslant C T(b-a) \Delta \max \limits _{j,p\in {\mathbb{N}}}\vert {\mathbf{u}}_j^p\vert . \end{aligned}$$

This tends to zero because $\max \limits _{j\in {\mathbb{Z}},p\in {\mathbb{N}}}\vert {\mathbf{u}}_j^p\vert$ is finite.

### **Lemma A3**

*Under the assumptions of Proposition* 1,

$$\begin{aligned} \lim \limits _{\Delta t, \Delta \rightarrow 0}\sum \limits _{n=0}^\infty \sum \limits _{[x_j, \,x_{j+1}], \, j\in {\mathbb{Z}}}\Delta t_n \varphi _{j+1/2} \delta _{j+1/2} {{\mathbf{f}}}=-\int _{{\mathbb{R}}\times {\mathbb{R}}^+} \frac{\partial \varphi }{\partial x}f(u)  {\mathrm{d}}x {\mathrm{d}}t. \end{aligned}$$

### **Proof**

This is again a simple adaptation of the classical proof since $\delta _{j+1/2}f=f(u_{j+1})-f(u_j)$. We have

$$\begin{aligned} \sum \limits _{[x_j, x_{j+1}], j\in {\mathbb{Z}}}\varphi _{j+1/2} \delta _{j+1/2} {{\mathbf{f}}}=-\sum \limits _{ j\in {\mathbb{Z}}}{{\mathbf{f}}}({\mathbf{u}}_j)\big( \varphi _{j+1/2}-\varphi _{j-1/2}\big) . \end{aligned}$$

Then using the boundedness of the solution and Lebesgue dominated convergence theorem, we get the result.

Then we have

### **Lemma A4**

*Under the conditions of Proposition* 1, *we have*

$$\begin{aligned} \lim \limits _{\Delta t, \Delta \rightarrow 0}\bigg(\sum \limits _{n\in {\mathbb{N}}}\sum \limits _{j\in {\mathbb{Z}}} \delta _{j}^{n+1/2} {\mathbf{u}}\bigg(\Delta _{j+1/2}\big( \varphi _j-\varphi _{j+1/2}\big) +\Delta _{j-1/2}\big(\varphi _j-\varphi _{j-1/2}\big)\bigg)\bigg)=0. \end{aligned}$$

### **Proof**

Since $\varphi \in C_0^\infty ({\mathbb{R}}\times {\mathbb{R}}^+)$, there exists *C* that depends only on the first derivative of $\varphi$ such that

$$\begin{aligned} \vert \Delta _{j+1/2}\big( \varphi _j-\varphi _{j+1/2}\big) +\Delta _{j-1/2}\big(\varphi _j-\varphi _{j-1/2}\big)\vert \leqslant C \Delta \; (\Delta _{j+1/2}+\Delta _{j-1/2}). \end{aligned}$$

Then using (10), we get

$$\begin{aligned} \delta _j^{n+1/2}:={\mathbf{u}}_j^{n+1}-{\mathbf{u}}_j^n=-\frac{\Delta t_n}{\Delta _j } \delta _x{\mathbf{u}}_{j}, \end{aligned}$$

and also

$$\begin{aligned} &\left\| \sum \limits _{j\in {\mathbb{Z}}} \delta _{j}^{n+1/2} {\mathbf{u}}\bigg(\Delta _{j+1/2}\big( \varphi _j-\varphi _{j+1/2}\big) +\Delta _{j-1/2}\big(\varphi _j-\varphi _{j-1/2}\big)\bigg)\right\| \\ & \leqslant C \Delta t_n\sum \limits _{j\in {\mathbb{Z}}} \Vert \delta _{x}^{n+1/2} {\mathbf{u}}\Vert \Delta _j\\ & =C \Delta \Delta t_n\sum \limits _{j\in {\mathbb{Z}}} \Vert \delta _x{\mathbf{u}}_{j}\Vert =C\Delta \sum \limits _{j\in \mathbb{Z}}\sum \limits _{l=-p}^{l=p}\vert {\mathbf{u}}_{j+l}-\bar{\mathbf{u}}_{j+l+1/2}\vert \end{aligned}$$

by using the Lipschitz continuity of the fluctuations and the regularity of the transformation ${\mathbf{v}}\mapsto {\mathbf{u}}$ together with the boundedness of the solution. Then, from Lemma A1, we see that the results hold true.

This ends the proof of Proposition 1.

## Appendix B Linear Stability Analysis

The scheme writes, setting $\lambda =a\frac{\Delta t}{\Delta x}$ and assuming $a>0$,

$$\begin{aligned} u_{j}^{n+1}&=u^n_j-2\lambda \delta _ju^n,\\ {\bar{u}}_{j+1/2}^{n+1}&={\bar{u}}_{j+1/2}^n-\lambda \big( u_{j+1}^n-u_j^n\big) \end{aligned}$$

on with periodicity 1. We set $\Delta x=\frac{1}{N}$. It is more convenient to work with point values only, and we will use the form

$$\begin{aligned} u_{j}^{n+1}&=u^n_j-2\lambda \delta _ju^n,\\ u_{j+1/2}^{n+1}&=u_{j+1/2}^n -\lambda \bigg( \frac{3}{2}\big( u_{j+1}^n-u_j^n\big)-\frac{1}{4}\big( \delta _j u^n+\delta _{j+1}u^n\big)\bigg). \end{aligned}$$

We perform a linear stability analysis by Fourier analysis. What is not completely standard is that the grid points do not play the same role. For ease of notations, we double the indices, this avoids to have half integer in the Fourier analysis. In other points, the quantities $u_j$ associated to the grid points $x_j$ are denoted by $u_{2j}$: this will be the even terms. Those associated to the intervals $[x_j,x_{j+1}]$, i.e., ${\bar{u}}_{j+1/2}$ and $u_{j+1/2}$ will be denoted as ${\bar{u}}_{2j+1}$ and $u_{2j+1}$, they are the odd terms, so that we use

A2 $$\left \{ \begin{aligned} u_{2j}^{n+1}&=u^n_j-2\lambda \delta _{2j}u^n,\\ u_{2j+1}^{n+1}&=u_{2j+1}^n -\lambda \bigg( \frac{3}{2}\big( u_{2j+2}^n-u_{2j}^n\big)-\frac{1}{4}\big( \delta _{2j}u^n+\delta _{2j+2}u^n\big)\bigg). \end{aligned}\right.$$

We have the Parseval equality,

$$\begin{aligned} \frac{1}{2N}\sum \limits _{j=0}^{2N-1} u_j^2=\sum _{k=0}^{2N-1} \vert {\hat{u}}(k)\vert ^2 \end{aligned}$$

with

$$\begin{aligned} {\hat{u}}(k)=\frac{1}{2N}\sum \limits _{j=0}^{2N-1} u_j{\text e}^{2i{\uppi} \frac{kj}{2N}}=\frac{1}{2}\big( {\hat{u}}_{\text o}(k)+{\hat{u}}_{\text e}(k)\big), \end{aligned}$$

where, by setting $\omega ={\text e}^{\frac{i\uppi }{N}},$

$$\begin{aligned} {\hat{u}}_{\text o}(k)=\frac{1}{N}\sum \limits _{j=0}^{N-1} u_{2j+1} \omega ^{(2j+1)k}, \quad {\hat{u}}_{\text e}(k)=\frac{1}{N}\sum \limits _{j=0}^{N-1} u_{2j} \omega ^{(2j)k}. \end{aligned}$$

The usual shift operator $[S(u)]_j=u_{j+1}$ gives

$$\begin{aligned} {S(\hat{u})}_{\text o}=\omega ^{-k} {\hat{u}}_{\text  e}, \quad {S(\hat{u})}_{\text  e}=\omega ^{-k} {\hat{u}}_{\text o}. \end{aligned}$$

Using this, we see that the Euler forward method (A2) gives

A3 $$\begin{aligned} \left(\begin{array}{c} {\hat{u}}_{\text o}^{n+1}\\ {\hat{u}}_{\text e}^{n+1}\end{array}\right)=\bigg( \text{Id}-\lambda H\bigg) \left(\begin{array}{c} {\hat{u}}_{\text o}^{n}\\ {\hat{u}}_{\text e}^{n}\end{array}\right) \end{aligned}$$

with

$$\begin{aligned} H_1(k)=\left(\begin{array}{cc} \frac{1+\omega ^{2k}}{4}& \frac{5\omega ^{-k}+7\omega ^k}{4}\\ 0& 2\big(1-\omega ^k\big)\end{array}\right) \end{aligned}$$

for the first order in space scheme,

$$\begin{aligned} H_2(k)=\left( \begin{array}{cc} \frac{1+\omega ^{2k}}{2} & \frac{9}{8}\omega ^{-k}-2\omega ^{-k}-\frac{\omega ^{3k}}{8}\\ 0& 2\big(1-\omega ^k\big)\end{array}\right) \end{aligned}$$

for the second order scheme, and

$$\begin{aligned} H_3(k) =\left( \begin{array}{cc} \frac{\omega ^{-2k}}{12}+\frac{1}{24}+\frac{\omega ^{2k}}{12}-\frac{\omega ^{4k}}{24} & \frac{31}{24}\omega ^{-k}-\frac{3}{2}\omega ^k+\frac{\omega ^{2k}}{12}+\frac{5}{24}\omega ^{3k}\\ 0& 2\big(1-\omega ^k\big)\end{array}\right) \end{aligned}$$

for the third order in time space.

Combined with the RK time stepping we end up with an update of the form

$$\begin{aligned} \left(\begin{array}{c} {\hat{u}}_{\text o}^{n+1}\\ {\hat{u}}_{\text e}^{n+1}\end{array}\right)=G_k\left(\begin{array}{c} {\hat{u}}_{\text o}^{n}\\ {\hat{u}}_{\text e}^{n}\end{array}\right), \end{aligned}$$

and writing

$$\begin{aligned} G_k=\left(\begin{array}{cc}\alpha _k & \beta _k\\ \gamma _k & \delta _k \end{array}\right) \end{aligned}$$

we end up with

$$\begin{aligned} {\hat{u}}_{\text o}^{n+1}= & {} \alpha _k {\hat{u}}_{\text o}^{n}+\beta _k {\hat{u}}_{\text e}^{n},\\ {\hat{u}}_{\text e}^{n+1}= & {} \gamma _k {\hat{u}}_{\text o}^{n}+\delta _k {\hat{u}}_{\text e}^{n} ,\end{aligned}$$

so that

$$\begin{aligned} {\hat{u}}^{n+1}=\frac{\alpha _k+\gamma _k}{2}{\hat{u}}_{\text o}^n+ \frac{\beta _k+\delta _k}{2}{\hat{u}}_{\text e}^n, \end{aligned}$$

from which we get

$$\begin{aligned} \vert {\hat{u}}^{n+1}\vert ^2=\frac{1}{4}\overline{ \left(\begin{array}{cc} {\hat{u}}_{\text o}^n&{\hat{u}}_{\text e}^n\end{array}\right) } M_k \left(\begin{array}{c} {\hat{u}}_{\text o}^n \\ {\hat{u}}_{\text e}^n\end{array}\right) \end{aligned}$$

with

$$\begin{aligned} M_k= \frac{1}{4}\left(\begin{array}{cc} \vert \alpha _k+\gamma _k\vert ^2 & \big(\alpha _k+\gamma _k\big)\overline{ \big(\beta _k+\delta _k \big)}\\ \overline{ \big(\alpha _k+\gamma _k\big)} { \big(\beta _k+\delta _k \big)} & \vert \beta _k+\delta _k \vert ^2\end{array}\right). \end{aligned}$$

We have the stability if the spectral radius of these matrices is always $\leqslant 1$, and we immediately see that

$$\begin{aligned} \rho (M_k)=\frac{1}{4}\bigg( \vert \alpha _k+\gamma _k\vert ^2+\vert \beta _k+\delta _k \vert ^2\bigg). \end{aligned}$$

After calculations, we see that the stability limits are

- first-order scheme, $\vert \lambda \vert \leqslant 0.92$,
- second-order scheme, $\vert \lambda \vert \leqslant 0.6$,
- third-order scheme, $\vert \lambda \vert \leqslant 0.5$.

## Appendix C Some Numerical Results on Irregular Meshes

To support the theoretical analysis of the method, we have applied it on irregular meshes. The goal is to show that even here, one gets convergence of the solution to a weak solution that appears to be the right one. Since we use the same schemes, there is no hope to get anything but first order accuracy. Accuracy on irregular meshes will be the topic of future work. The mesh is defined by: for $0\leqslant i\leqslant N$,

$$\begin{aligned} y_0=0, \quad y_{i+1}=y_i+\Delta y, \quad \Delta y=\frac{1+\epsilon _i/2}{N} \end{aligned}$$

and

$$\begin{aligned} \epsilon _0=-1, \epsilon _{i+1}=-\epsilon _i. \end{aligned}$$

Then we define the actual mesh by

$$\begin{aligned} x_i=\frac{y_i}{y_N}. \end{aligned}$$

On the Sod problem, with $N=10{\,}000$, we get the results of the Fig. A1.

In Fig. A2, we show a zoom of the density around the shock wave. The discretisation points as well as the numerical and exact solutions are shown.

## Footnotes

1 The author works on $\frac{\partial u}{\partial t}=-\frac{\partial u}{\partial x}$ which is a bit confusing w.r.t. to “modern” habits. It is true that British drive left.
